# Comparative Analysis of Plasmodium falciparum Genotyping via SNP Detection, Microsatellite Profiling, and Whole-Genome Sequencing

**DOI:** 10.1128/AAC.01163-21

**Published:** 2022-01-18

**Authors:** Mariko Kanai, Tomas Yeo, Victor Asua, Philip J. Rosenthal, David A. Fidock, Sachel Mok

**Affiliations:** a Department of Microbiology and Immunology, Columbia University grid.21729.3fgrid.239585.0 Irving Medical Center, New York, New York, USA; b Center for Malaria Therapeutics and Antimicrobial Resistance, Division of Infectious Diseases, Department of Medicine, Columbia University grid.21729.3fgrid.239585.0 Irving Medical Center, New York, New York, USA; c Infectious Disease Research Collaboration, Kampala, Uganda; d Department of Medicine, University of California, San Francisco, California, USA

**Keywords:** *Plasmodium falciparum*, drug resistance, genotyping, malaria, microsatellites

## Abstract

Research efforts to combat antimalarial drug resistance rely on quick, robust, and sensitive methods to genetically characterize Plasmodium falciparum parasites. We developed a single-nucleotide polymorphism (SNP)-based genotyping method that can assess 33 drug resistance-conferring SNPs in *dhfr, dhps, pfmdr1, pfcrt,* and *k13* in nine PCRs, performed directly from P. falciparum cultures or infected blood. We also optimized multiplexed fragment analysis and gel electrophoresis-based microsatellite typing methods using a set of five markers that can distinguish 12 laboratory strains of diverse geographical and temporal origin. We demonstrate how these methods can be applied to screen for the multidrug-resistant KEL1/PLA1/PfPailin (KelPP) lineage that has been sweeping across the Greater Mekong Subregion, verify parasite *in vitro* SNP-editing, identify novel recombinant genetic cross progeny, or cluster strains to infer their geographical origins. Results were compared with Illumina-based whole-genome sequence analysis that provides the most detailed sequence information but is cost-prohibitive. These adaptable, simple, and inexpensive methods can be easily implemented into routine genotyping of P. falciparum parasites in both laboratory and field settings.

## INTRODUCTION

In 2019, there were an estimated 229 million malaria cases and 409,000 deaths, spanning 87 malaria-endemic countries, with most deaths resulting from Plasmodium falciparum infections in African children (1). The emergence and spread of multidrug-resistant P. falciparum strains complicate efforts to reduce malaria morbidity and mortality. Crucial components of malaria research include tracking the emergence of drug-resistant parasites, identifying and investigating genetic markers that correlate with treatment failure, and discovering effective antimalarials with novel modes of action.

These research initiatives have incentivized a long-standing interest in developing robust, sensitive, and cost-effective genotyping methods. Accuracy is important to ensure correct strain identification, and sensitivity is essential for assessing clonality by detecting lower frequency alleles such as those found in mixed infections or cross-contaminated lab cultures. Even a 10% contamination of a drug-sensitive strain with a drug-resistant strain can impact drug susceptibilities (2, 3). Cost-effectiveness is also necessary to ensure routine use. These methods should also allow the study of parasitized blood dried on filter paper, a routine means of storing samples in the field.

Historically, P. falciparum parasites were genotyped by assessing length- and sequence-polymorphic molecular markers such as the merozoite surface proteins *msp1* and *msp2* and the glutamine rich protein *glurp* (4–9). These markers can distinguish new infections from recrudescence, and help assess treatment efficacy (10). These three genes, however, are under host immune selective pressure and their analysis has been shown to underestimate treatment failure rates and insufficiently capture parasite genetic diversity (11). Thus, microsatellites and single-nucleotide polymorphisms (SNPs) are commonly used for genotyping parasites, and these analyses can be complemented by whole-genome sequencing (WGS) (12).

Microsatellites form when the DNA replication complex slips off the template strand of one repeat unit, and re-attaches at a different unit, creating “errors” or new strands with longer or shorter sequences (13). In P. falciparum, microsatellites are mostly (TA)_n_ or (T or A)_n_, ranging from 10 to 30 repeats (14). SNPs most commonly form due to errors during DNA replication and after environmental exposures to DNA-damaging agents. Nonsynonymous SNPs or copy number variations (CNVs) can mediate resistance through amino acid substitutions and/or expression level changes in resistance determinants (15). Approaches to identify resistance determinants include molecular genotyping, genome-wide association studies, and positional mapping using genetic cross progeny (16–18).

Resistance to quinine, a drug used to treat severe malaria, has been partially associated with SNPs in the P. falciparum chloroquine resistance transporter (*pfcrt*) and P. falciparum multidrug resistance-1 (*pfmdr1*) genes (19–21). Resistance to chloroquine (CQ), the former front-line antimalarial, is mediated primarily by polymorphisms in PfCRT, of which K76T is critical, and can be modulated by polymorphisms in PfMDR1 (22–24). Novel SNPs encoding for mutations in PfCRT (including T93S, H97Y, F145I, I218F, M343L, and G353V) that emerged on the CQ-resistant Dd2 PfCRT variant, as well as amplifications in plasmepsins (*pm*) 2 and 3 genes, were recently shown to be the primary mediators of resistance to piperaquine (PPQ), a partner drug used in artemisinin (ART)-combination therapy (ACT) (25–27). PfMDR1 N86Y and D1246Y, as well as select PfCRT variants, have also been associated with *in vitro* and *in vivo* resistance to amodiaquine, another ACT partner drug (24, 28–31). Increased *pfmdr1* copy number *in vitro* and *in vivo* has been associated with low-grade resistance to mefloquine, which is partnered with artesunate (1, 32, 33). SNPs encoding for mutations in the folate biosynthesis enzymes dihydrofolate reductase (DHFR) (N51I, C59R, S108N, I164L) and dihydropteroate synthase (DHPS) (A437G, K540E) confer resistance to pyrimethamine and sulfadoxine, respectively (15, 34). DHPS A581G associates with enhanced *in vitro* resistance to both drugs (35).

Delayed parasite clearance times after treatment with the current front-line antimalarial, ART, and its derivatives are driven by mutations in the Kelch13 (K13) propeller domain, of which C580Y dominates in the Greater Mekong Subregion (GMS) (36–38). Isolates harboring C580Y have also been observed in Guyana and Papua New Guinea (39, 40). ART resistance-conferring K13 mutations in the GMS (including R539T and C580Y) are not yet prevalent in Africa, although the emergence of the ART resistance-conferring R561H mutation in Rwanda and recent evidence that it can confer resistance *in vitro* raises important concerns (41–45). This worsening situation indicates an urgent need to monitor *k13* as well as genetic markers of resistance to ACT partner drugs.

Microsatellites have also been useful for studying evolution, migration, and interrelatedness between strains, including analysis of linkage disequilibrium and selective sweeps in drug-resistant P. falciparum isolates (46). Microsatellite genotyping has revealed a common genetic signature surrounding the K13 C580Y variant (PfPailin lineage) in a majority of isolates across the GMS, indicating a transnational ART selective sweep that originated in Cambodia (47). This PfPailin lineage subsequently gave rise to the KEL1 (K13 C580Y)/PLA1 (multicopy *pm2-3*) co-lineage, which more recently includes new PfCRT variants and which is primarily responsible for DHA-PPQ treatment failure in the GMS (48–53). Sets of 12 microsatellite markers have also been used to genotype P. falciparum clinical isolates from finger-prick blood samples or to distinguish genetic cross progeny (54–56).

Here, we provide rapid and inexpensive tools for classifying strains, identifying drug resistance haplotypes, and assessing clonality. We describe a rapid and adaptable method to genotype SNPs from cultured parasites or infected blood, and compare this platform with optimized genotyping using either whole-genome sequencing or microsatellite markers that can distinguish geographically diverse laboratory strains.

## RESULTS

### PCR-based SNP genotyping from parasite cultures.

**FIG 1:**
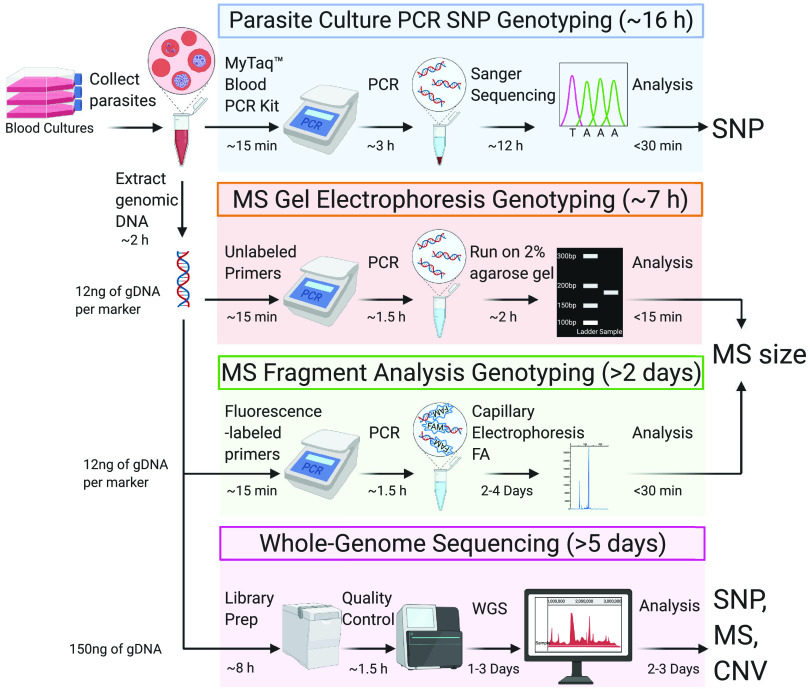
Workflow of the single-nucleotide polymorphism (SNP) and microsatellite (MS) genotyping methods described herein. SNPs can be identified using PCR and Sanger sequencing of parasite cultures or whole-genome sequencing (WGS) of purified parasite DNA. PCR-based SNP genotyping utilizes asexual blood stage cultures whereas MS genotyping and WGS require genomic DNA. MS sizes can be identified visually by gel electrophoresis or quantitatively by fragment analysis (FA) or by WGS. FA can be conducted on a DNA analyzer such as the ABI3730xl or SeqStudio Genetic Analyzer (Applied Biosystems). WGS requires a quality control step using the Bioanalyzer or TapeStation systems (Agilent). Protocol durations are for groups of up to ∼24 samples and minimum amounts of DNA typically used per protocol are indicated. Image was created with BioRender.com (refer to Tables S1 and S2 and the supplemental methods).

**FIG 2:**
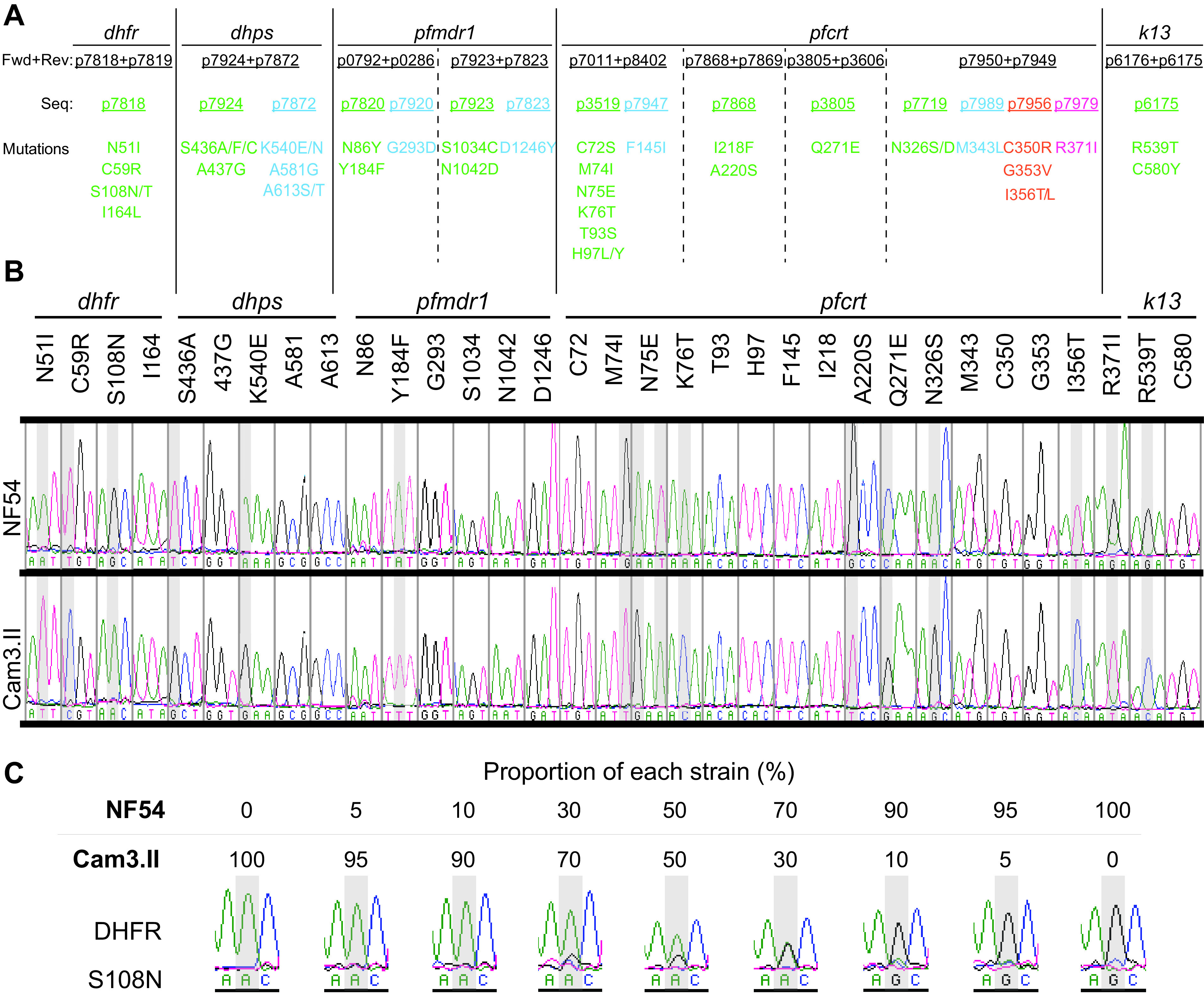
Parasite culture-based PCR genotyping to assess 33 single-nucleotide polymorphisms (SNPs) in *dhfr*, *dhps*, *pfmdr1*, *pfcrt*, and *k13* (see Table S1 and the supplemental methods). (A) Amino acid mutations in these drug resistance determinants can be assessed by each PCR (separated by vertical lines) and are listed with forward, reverse, and sequencing primers that are color-coded by the sequencing primer. (B) Sanger sequencing electropherograms of these amino acid positions for NF54 and Cam3.II parasites. (C) Sensitivity test results of the parasite culture PCR/Sanger sequencing method for the *dhfr* genotype in nine mixtures containing defined ratios of NF54 and Cam3.II cultures (see Fig. S1 for other positions). Representative electropherograms showing S108 (NF54) or S108N (Cam3.II) alleles.

We selected a panel of 12 well-characterized laboratory-adapted P. falciparum strains representing diverse genomes, drug susceptibility profiles, and geographic origins, and used next-generation sequencing to analyze their genomes (Table 1; Fig. S4C). We then developed PCR and Sanger sequencing reaction protocols that can test directly from asexual blood stage parasites, without a genomic DNA (gDNA) extraction step, and that can be completed in less than a day (Fig. 1). Our nine PCR conditions and 16 Sanger sequencing reactions cover 33 amino acid changes in five genes associated with drug resistance: *dhfr*, *dhps*, *pfmdr1*, *pfcrt*, and *k13* (Fig. 2A to C; Table S1). Primers and PCR conditions are listed in Table S1, and the protocol is listed in the supplemental methods. All 33 variant codons were assessed in six strains: NF54, Cam3.II, RF7, GB4, Dd2, and 7G8 (Fig. 2B and C). All of the amino acid sequences determined by PCR/Sanger sequencing correctly matched the whole-genome sequence data, validating our methods.

**TABLE 1:**
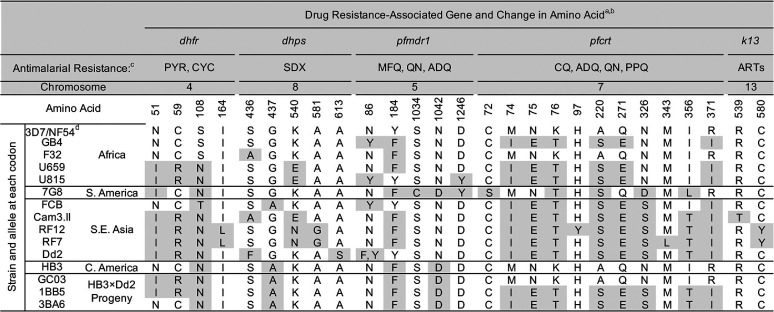
All single-nucleotide polymorphisms (SNPs) and their corresponding amino acid mutations assessed by PCR/Sanger sequencing of parasite cultures for a panel of 15 P. falciparum strains

a Six other SNPs can be assessed by our parasite culture PCR conditions but are not listed in this table, as all 15 strains have the wild-type allele: (*pfmdr1* G293, and *pfcrt* T93, F145, I218, C350, G353).

b Gene IDs are *dhfr* (PF3D7_0417200), *dhps* (PF3D7_0810800), *pfmdr1* (PF3D7_0523000), *pfcrt* (PF3D7_0709000), and *k13* (PF3D7_1343700).

c The full names for the antimalarials are PYR (pyrimethamine), CYC (cycloguanil), SDX (sulfadoxine), MFQ (mefloquine), QN (quinine), ADQ (amodiaquine), CQ (chloroquine), PPQ (piperaquine), and ARTs (artemisinins).

d 3D7 is a clone of NF54; as such, these are considered to be the same strain.

To assess sensitivity, we assayed nine mixtures of synchronized blood stage cultures of NF54 and Cam3.II strains at different ratios (0:100; 5:95; 10:90; 30:70; 50:50; 70:30; 90:10; 95:5; 100:0) and visually examined the electropherograms (Fig. 2C; Fig. S1). PCR/Sanger sequencing was sensitive up to a 70:30 or 90:10 polyclonal mix, with sensitivity as high as 95:5 in one instance. This finding suggests that PCR/Sanger sequencing is useful for quickly validating strains and profiling drug resistance markers, but has limited ability to detect minor alleles in a mixed sample.

### Evaluation of microsatellite-based gel electrophoresis and fragment analysis.

We next assessed microsatellite-based genotyping, and identified a set of five microsatellite markers (Table 2) that uniquely identify our panel of 12 P. falciparum strains (Fig. 1). Markers were selected from a published list (56) based on their distribution on distinct chromosomes, size difference relative to 3D7 (mostly ≥ 6 bp), and the minimum set of markers required to distinguish these strains. For this PCR-based approach, we compared agarose gel electrophoresis with capillary-based fragment analysis (FA). We also optimized microsatellite typing methods and assessed their resolution and sensitivity (see Table S2 and the supplemental methods). The KAPA HiFi DNA polymerase (Roche) was chosen due to its documented low error rate and efficient amplification across highly AT-rich genomes (57).

**TABLE 2:**
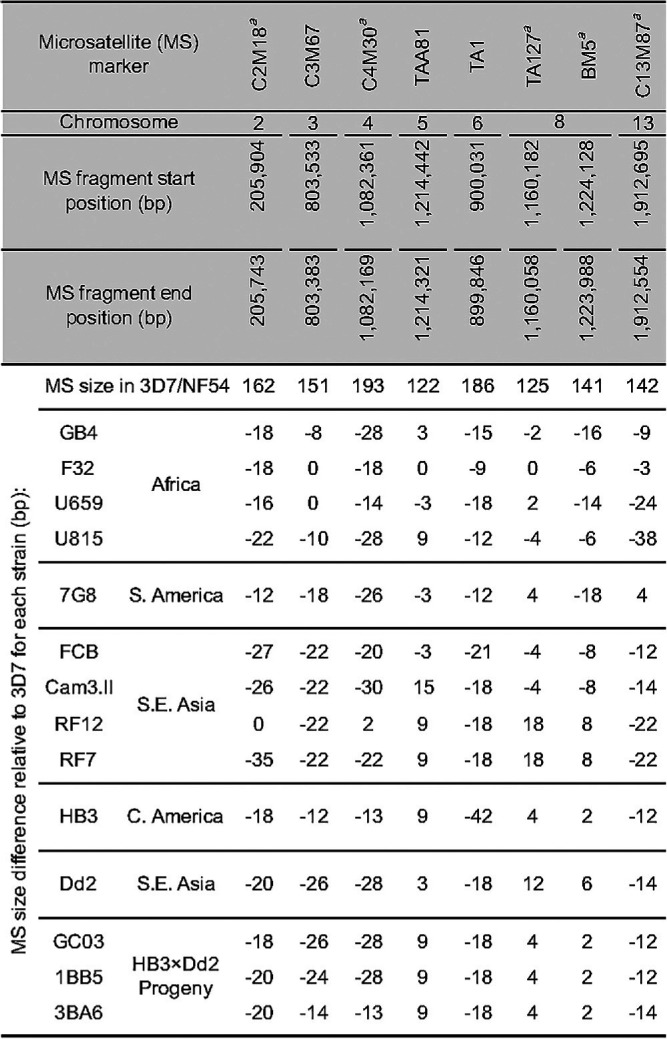
Microsatellite sizes determined from whole-genome sequencing (WGS) of the 15 P. falciparum strains assayed herein

a Indicates the five MS markers selected for genotyping laboratory strains.

**FIG 3:**
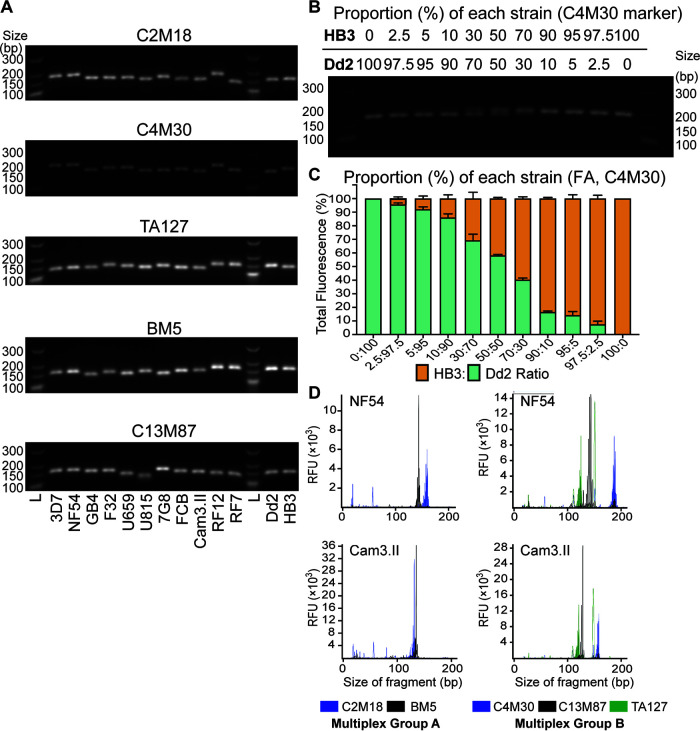
Selection of five microsatellite (MS) markers to distinguish 12 P. falciparum strains by gel electrophoresis or fragment analysis (FA) (see Table 2). (A) Gel electrophoresis image capturing the MS PCR product sizes that vary between strains. (B) Sensitivity of gel electrophoresis MS genotyping for C4M30 tested in 11 mixtures containing different ratios of HB3:Dd2 genomic DNAs (see Fig. S2 for remaining markers). (C) Sensitivity of FA genotyping of C4M30 in these mixtures to determine the proportion of HB3 and Dd2 fragment sizes. Bars represent the average fragment size determined from the fluorescence signals in two experiments. Error bars represent SEM values. L, ladder. (D) FA electropherograms obtained from fluorescently labeled PCR products multiplexed in two groups for NF54 and Cam3.II parasites. FAM, fluorescein amidite; RFU, relative fluorescence unit.

For each strain, we observed expected size differences relative to the 3D7 reference genome (Fig. 3A; Fig. S2A). However, for the FA method, the absolute sizes of the microsatellite products varied occasionally between runs. Thus, we recommend that for both methods a reference strain (e.g., 3D7) should be included in every run. The correlation of the relative microsatellite size from FA versus WGS yielded a mean R^2^ = 0.98 (Fig. S2B) across these five microsatellite markers. Gel electrophoresis had a minimum resolution of 9 bp difference between strains, while FA was accurate to within a 2 bp difference.

We also assessed the sensitivity of gel electrophoresis-based and FA-based microsatellite genotyping. This was tested with 11 mixtures containing different ratios of HB3:Dd2 input gDNA (0:100; 2.5:97.5; 5:95; 10:90; 30:70; 50:50; 70:30; 90:10; 95:5; 97.5:2.5; 100:0). Mixtures were genotyped at the five microsatellite markers (Fig. 3B and C; Fig. S3A and B). FA-based detection of the minor population was sensitive to as low as 2.5% for all microsatellite markers, whereas gel electrophoresis could only detect down to 10% for C4M30, C3M67, and TA1; and 30% for TAA81 and TA127. To increase throughput, we also multiplexed FA-based microsatellite genotyping. Forward primers were fluorescently labeled with 6-FAM (blue) and ATTO550/NED (yellow) and used in two separate PCRs for C2M18 and BM5, respectively, for each strain (Fig. 3D; Multiplex Group A). We also labeled forward primers with 6-FAM, ATO550, and ATTO532/VIC (green) for C4M30, C13M87, and TA127, respectively (Multiplex Group B). PCR products were mixed at either a 2:1 ratio for Multiplex Group A or at a 1:1:1 ratio for Multiplex Group B. These multiplex runs showed 100% accuracy for the five markers.

### Classification of genetic cross progeny by whole-genome sequencing versus fragment analysis-based microsatellite genotyping.

**FIG 4:**
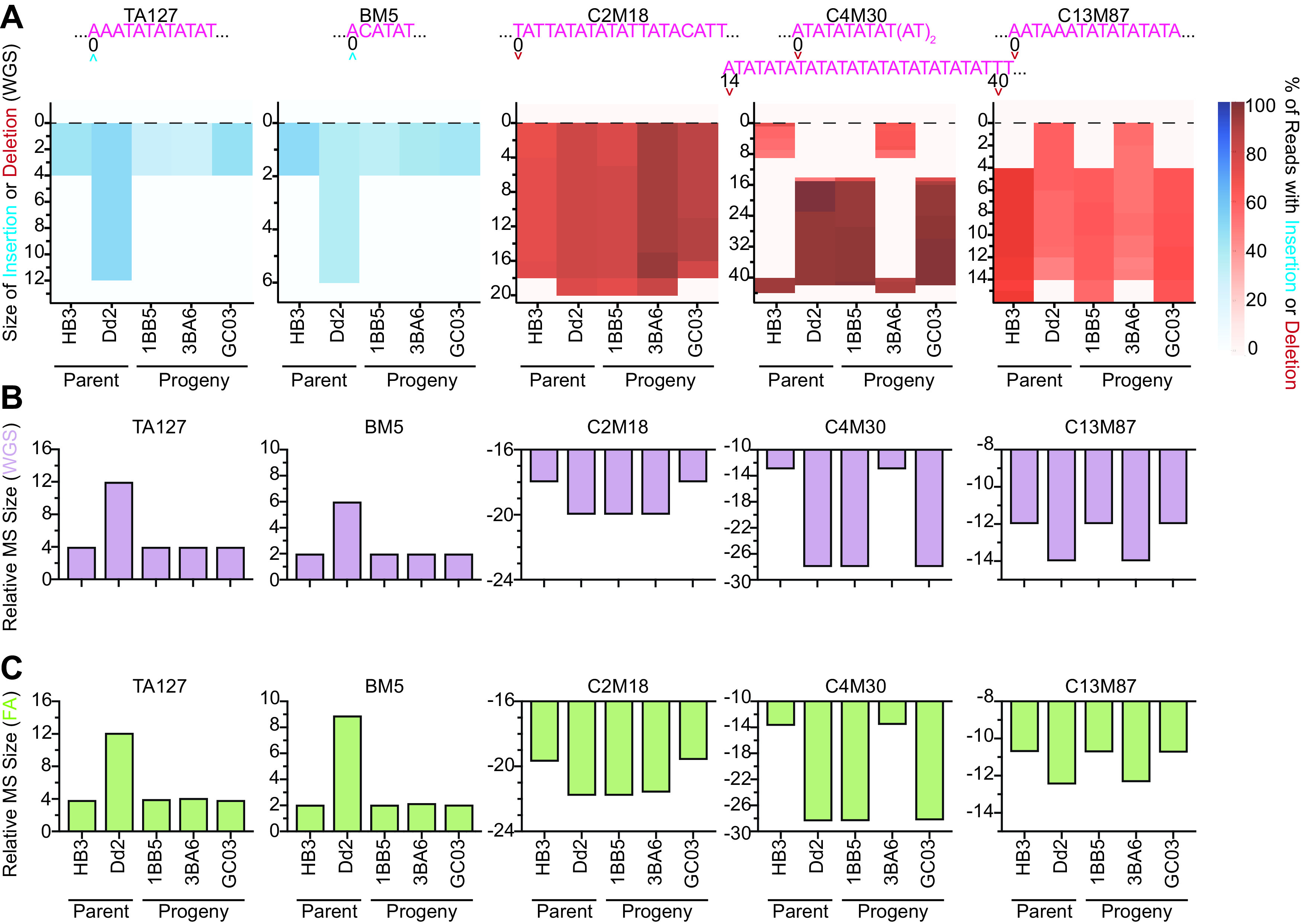
Comparison of microsatellite (MS) typing by fragment analysis (FA) and whole-genome sequencing (WGS) analysis to classify parents and progeny of the HB3×Dd2 genetic cross. (A) Heatmap of the WGS MS typing data indicating the fragment insertion or deletion size for each parasite relative to 3D7. The MS sequence for 3D7 (pink) and the position at which the insertion or deletion occurred within the MS fragment window are shown. The percentage of sequence reads covering the MS region that have base insertions or deletions are shown by the blue or red scale, respectively. The net MS sizes relative to the 3D7 reference are shown from (B) WGS-based or (C) FA-based genotyping methods.

SNP and microsatellite genotyping methods from parasitized blood are less expensive, faster, and more accessible than WGS. In addition to characterizing lab strains, microsatellite genotyping can also be used to classify P. falciparum genetic cross progeny without existing WGS data (Fig. 4). This is of increasing relevance given the recent availability of humanized mouse models to generate genetic crosses (58), and the need to ensure clonal integrity during continuous cell culture. To assess feasibility, we used our five microsatellite markers to genotype parents of the HB3×Dd2 genetic cross and its representative progeny 1BB5, 3BA6, and GC03 (59). From the WGS data, heatmaps were generated to identify the location and length of microsatellite insertions and/or deletions, relative to the 3D7 reference (Fig. 4A and B). Microsatellite sizes relative to 3D7 were also obtained from the FA data (Fig. 4C). Results correctly assigned each microsatellite marker in the progeny to parental HB3 or Dd2 origins, consistent with WGS data for the progeny. Thus, in addition to ensuring clonal integrity of cross progeny clones, FA typing with microsatellite markers can also be used to screen for recombinant progeny as long as they are polymorphic between the genetic cross parents.

### Application of SNP and microsatellite genotyping approaches to clinical samples.

We tested whether our optimized PCR/Sanger sequencing methods using parasite cultures could be applied to simulated fresh and frozen whole-blood samples, as well as dried blood spots (DBSs), by using P. falciparum laboratory cultures mixed with whole-blood (final hematocrit 40–50%) (Table S3). While PCR/Sanger sequencing using simulated fresh and frozen whole-blood samples were able to detect *dhfr*, *dhps*, *pfmdr1*, *pfcrt*, and *k13* down to 0.4% parasitemia, those using gDNA extracted from the DBSs were the most robust, with sensitivity down to 0.02% parasitemia.

We further investigated the applicability of these genotyping methods using gDNA extracted from DBSs collected from 16 symptomatic malaria patients in eastern Uganda in 2019 (Table S4). We were able to robustly determine the drug resistance alleles for all five genes. Almost all samples had multiple mutations in DHFR (N51I/C59R/S108N) and DHPS (K540E/A437G), 6/16 were pure PfMDR1 Y184F mutants (two more were mixed infections), and one sample was a PfCRT K76T mutant. No samples harbored any nonsynonymous mutation in the K13 beta-propeller domain. We also characterized these samples using microsatellite genotyping and fragment analysis. Results demonstrated greater genetic diversity among parasites using microsatellites compared to SNP genotyping. PCR/Sanger sequencing and FA analysis identified polyclonal infections in six and nine samples, respectively (Table S4).

### SNP versus microsatellite-based clustering of geographically diverse parasites.

**FIG 5:**
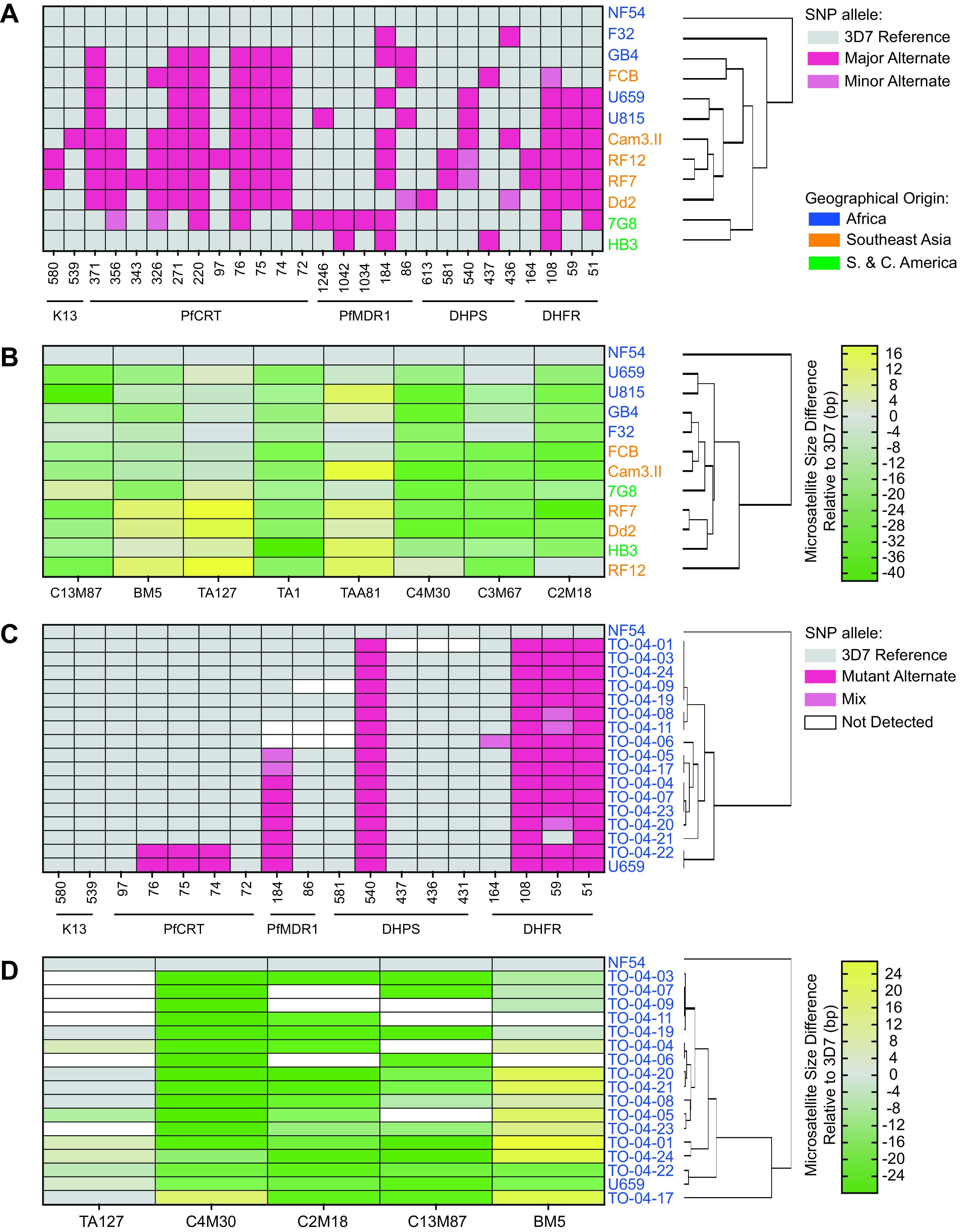
Hierarchical clustering of laboratory strains and Ugandan dried blood spot samples by their respective geographical origins using microsatellite (MS) or single-nucleotide polymorphism (SNP) markers. (A) Heatmap showing the clustering of 12 laboratory lines using 27 discriminatory SNPs in *dhfr*, *dhps*, *pfmdr1*, *pfcrt*, and *k13*. The other six SNPs were identical across all strains tested. (B) Heatmap depicting these laboratory strains clustered by eight MS markers. Parasite geographical origins are indicated by font color. Major versus minor alternate alleles, and the size of MS insertions or deletions, are indicated by the color scale. (C) Heatmap showing the clustering of 16 Ugandan samples alongside two control lines using 18 SNPs in *dhfr*, *dhps*, *pfmdr1*, *pfcrt*, and *k13*. (D) Heatmap of Ugandan samples and two control lines clustered by five MS markers (also see Table S4).

We also assessed whether 27 discriminatory SNPs in drug resistance genes or eight microsatellite markers could segregate our set of 12 laboratory strains according to their geographical origin (Fig. 5A and B). Both SNP and microsatellite genotyping approaches grouped the African strains. The 7G8 and HB3 strains from South and Central America, respectively, segregated correctly only with the SNP method. They did, however, consistently segregate closer to Southeast Asian lines than to African lines with both methods. Overall, these data suggest that the drug resistance-conferring SNPs and a small set of microsatellite markers can broadly segregate parasites by continent.

To identify clades within the Ugandan clinical samples, we also conducted SNP- and microsatellite-based clustering (Fig. 5C and D). Using SNPs, Ugandan parasites clustered into three major groups segregating by *pfmdr1* and *pfcrt* status, whereas microsatellite clustering revealed five major groups. Interestingly, TO-04-22 appeared identical to U659, another Ugandan parasite isolated in 2007, by both SNP and microsatellite clustering, suggesting a shared ancestry.

### Integrated analysis of KEL1/PLA1/PfPailin (KelPP) status.

**FIG 6:**
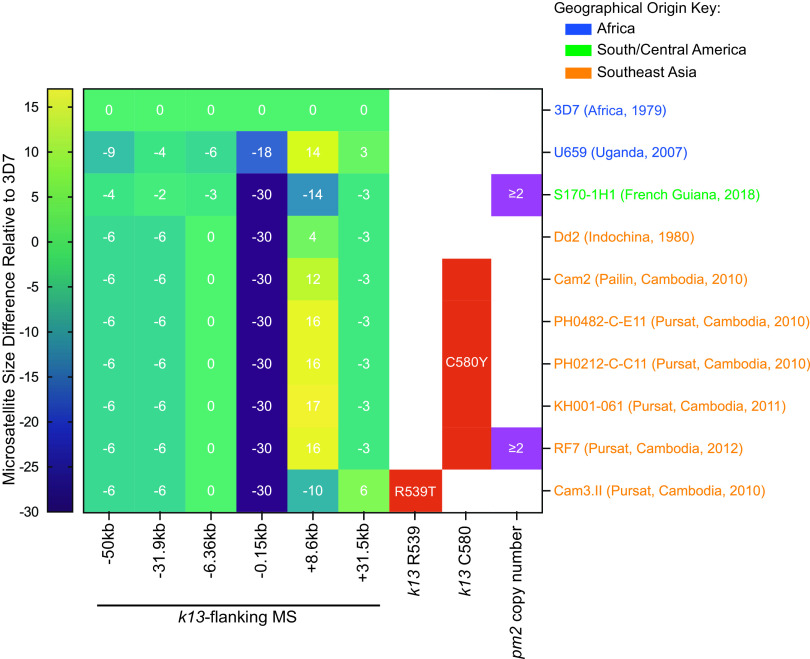
Testing for the KEL1/PLA1/PfPailin (KelPP) co-lineage in 10 geographically diverse P. falciparum strains, based on *kelch13* (*k13*)-flanking microsatellite sizes relative to 3D7, K13 mutations, and *plasmepsin 2* (*pm2*) copy number (see Table S5 and the supplemental methods). The sizes of the *k13*-flanking microsatellite insertion or deletion are indicated by the color scale. RF7 was the sole KelPP strain in this set.

We also applied our *k13* SNP genotyping method, and previously published *k13*-flanking microsatellite genotyping and *pm2* copy number quantification methods (26, 47), to test whether parasites had a KelPP co-lineage haplotype associated with an increased risk of DHA-PPQ treatment failure (Fig. 6, Table S5, and the supplemental methods). As expected, the *k13*-flanking microsatellite sizes for African and South American strains differed substantially from those of the Cambodian strains. The latter all shared the same microsatellite sizes on the four upstream (–50 kb to −0.15 kb) markers, but differed at the +8.6 kb marker. Almost all sampled Cambodia isolates from 2010 onwards had mutant K13 C580Y, consistent with the emergence in ∼2008 of the multidrug-resistant PfPailin lineage (47). RF7 was the only Cambodian strain to have both multicopy *pm2* and mutant K13 and corresponds herein to the KelPP haplotype. This finding is also consistent with the rapid replacement of the KEL1 lineage with the KEL1/PLA1 co-lineage in Cambodia (38, 51, 52, 60).

## DISCUSSION

Herein, we describe several methods to genotype P. falciparum parasites for a range of experimental needs that include validating parental or genetically modified lab strains, profiling new field isolates, or identifying genetic cross recombinant progeny. These methods include PCR/Sanger sequencing from parasite cultures, and optimization of a set of five polymorphic markers for multiplexed microsatellite genotyping. The PCR/Sanger sequencing-based SNP genotyping method, applied directly to parasite cultures, is quick and accurate, making it ideal to routinely validate lab strains and confirm gene editing in transfected parasites (Table 3). This approach can help profile drug resistance markers and identify known and novel mutations with culture-adapted isolates. Our genotyping was also optimized to identify the KEL1/PLA1/PfPailin (KelPP) co-lineage that has spread rapidly throughout the GMS. This PCR-based method of detecting K13 C580Y directly from P. falciparum-infected red blood cells (RBCs) can expedite the identification of ART-resistant clinical isolates, and help prioritize further screening for the KelPP co-lineage. However, our panel of SNP-based genotyping markers will not detect emerging and/or novel drug resistance mutations outside the amplified gene fragments. In contrast, WGS analysis provides comprehensive SNP genotyping that can capture novel mutations, elucidate genetic diversity, and provide insights into the selective pressures impacting parasite genomes (61, 62). However, WGS is cost-prohibitive, time-consuming, and requires specialized instrumentation and bioinformatic tools, limiting its adoption for routine genotyping.

**TABLE 3:** Comparison of the genotyping methods used in this study^*a*^

	Typing method	Resolution	Sensitivity	Time	Cost	Pros	Cons	Ideal application
SNP	Parasite culture PCR + Sanger sequencing	1 bp	≥10%	∼16 h	Moderately expensive^*b*^	Quick, accurate	Requires specialized equipment, genetic info only around SNP	Validate strains, drug resistance profiling, confirm gene-editing
MS	Gel electrophoresis	9 bp	≥10%	∼7 h	Inexpensive	Quick, no specialized equipment required	Lowest resolution, visual determination, genetic info only on MS	Validate strains, identify novel genetic cross progeny
MS	Fragment analysis	2 bp	≥2.5%	>2 days	Moderately expensive	Quantitative, moderate sensitivity	Requires specialized equipment, genetic info only on MS	Validate strains, detect cross-contamination, identify novel genetic cross progeny, infer origin
SNP + MS	WGS	1 bp	≥4%^*c*^	>5 days	Expensive	High sensitivity and accuracy, provides unbiased genotyping data	Expensive, time-consuming, requires specialized equipment and bioinformatics skills	Drug resistance profiling, detect contamination, identify novel genetic cross progeny, infer origin

a SNP, single-nucleotide polymorphism; MS, microsatellite; WGS, whole-genome sequencing.

b The cost per SNP using parasite culture PCR genotyping depends on the PCR reaction used.

c Assuming a mean fold coverage of 50× or greater.

Alternative TaqMan- or GoldenGate-based methods described by other groups utilize a custom panel of informative SNPs to create parasite “barcodes,” identify lineages, and explore external selection pressures and parasite transmission (9, 63–66). These customized methods, which require gDNA extraction, were designed to discriminate between two separate DNA templates differing at a predefined SNP position. TaqMan-based allelic discrimination was also applied to genotype *pfcrt* K76T directly from whole blood (67). Another approach employs a high-resolution melting analysis, in which <250 bp amplicons harboring SNP positions of interest are melted post-PCR, and variants are detected if the shape of the melt curves and *T_m_* values differ from those of the wild-type control (68, 69). While this method can detect the presence of more than one variant in each SNP position and several SNPs within a single amplicon, it requires Sanger sequencing to elucidate variants. Other genetic epidemiology tools include amplicon sequencing (70, 71), multiplexed mass spectrometry-based genotyping (71), high-throughput targeted sequencing using molecular inversion probes (72), and ligase detection reaction fluorescent microsphere assays (43). These methods, however, require gDNA extraction and specialized equipment. Amplicon sequencing and molecular inversion probes must be combined with next-generation sequencing while ligase detection reaction fluorescent microsphere assays can only detect specific alleles on a bead array laser scanner. Our method of SNP/Sanger sequencing directly from parasite cultures does not require gDNA extraction, can detect multiple alleles in the amplified fragment, is cost-effective, and is readily customizable. These attributes will be valuable in detecting novel K13 resistance-conferring mutations, especially in high-transmission regions in Africa (41). We show herein that this method can be successfully applied to define drug resistance genotypes directly from fresh or frozen parasite samples as well as gDNA extracted from DBSs from P. falciparum-infected patients.

Gel electrophoresis-based microsatellite genotyping can be readily implemented, is quick, inexpensive, and has sufficient accuracy and resolution for validating strains in the laboratory setting and/or in the field. This method is also amenable to identifying recombinant genetic cross progeny. However, given the low resolution and qualitative assessment, some strains will be difficult to genotype if the size difference between strains is <9 bp or if a minor population is <10%. We found the fluorescence-based capillary electrophoresis FA genotyping method to have a higher resolution (2 bp) and greater sensitivity (minor parasite population >2.5% of total). This method is also quantitative, making it suitable for detecting cross-contamination in a laboratory culture or polyclonal infections in field samples, as we showed with isolates from Uganda. Another advantage is that FA can be multiplexed either pre- or post-PCR to increase throughput. We recommend the latter as melting temperatures (T*_m_*) may vary between primers. Also, microsatellites are often laden with AT-repeats, making it difficult to design compatible primer pairs that lack complementarity. While microsatellites have been previously multiplexed by hemi-nested PCR to assess field isolates (55), these markers may not be as relevant for multiplexed genotyping of common lab strains. Microsatellite genotyping can also be conducted directly from asexual blood stage P. falciparum cultures, as shown previously (73).

Although less expensive and quicker than WGS, microsatellite genotyping is inferior to WGS in a few aspects. WGS genotyping does not need a positive control strain (e.g., 3D7) for each run, can identify the genomic location of microsatellite insertions and deletions, and can assess a much larger number of microsatellites (74). However, errors can be introduced in this method because of the similarity in length between Illumina reads and microsatellite arrays that can complicate sequence alignment, and homopolymers that can cause sequencing errors (75, 76).

On average, microsatellites account for 3% of the human genome and ∼11% of the P. falciparum genome (76, 77). P. falciparum is thought to have an especially high number of microsatellites in part due to the AT-rich genome (81% overall; 90% in noncoding regions) that may increase the chances of polymerase slippage and AT-repeat microsatellite formation (12). Across the P. falciparum genome, an average of 80% of insertions and deletions (indels) are short tandem repeats that include microsatellites, and 83% of indels are located in noncoding regions (78). The indel:SNP ratio is ∼3:1 and ∼1:2 in noncoding and coding regions, respectively, reflecting greater constraints on nucleotide diversity in genes. Our observation that drug resistance-conferring SNPs are subjected to stronger selective pressures than microsatellites was evident in the geographical segregation of parasite lines through our phylogenetic analyses using SNPs or microsatellite markers (Fig. 5).

Despite their seeming potential for increased mutability, microsatellites are relatively stable in P. falciparum. In an *in vitro* evolution experiment, the mutation rate for 3D7 P. falciparum microsatellites was on average 3.1 × 10^−7^ to 2.2 × 10^−8^ per cell division, with longer microsatellite array lengths and motifs correlating with higher mutation rates (76). This mutation rate is low when compared with those of other organisms including humans (∼10^−3^ per locus per gamete), Escherichia coli (∼10^−2^), Saccharomyces (∼10^−5^), and Drosophila (∼10^−6^ to 10^−4^) (79, 80), possibly because *Plasmodium* spp. lack the nonhomologous end-joining pathway during DNA repair, which may reduce replication errors during DNA damage repair (76, 81, 82). Nonetheless, we cannot disregard the possibility that microsatellite sizes may change over long-term *in vitro* culture (83).

The five microsatellite markers that we selected span 3′ untranslated regions (UTRs), or exon-UTR or exon-intron boundaries of essential genes (Table S2) (84). The repeat motifs in these microsatellite markers were mostly di- to tetra-nucleotide repeats (Table S2). As larger motif sizes are associated with higher microsatellite mutation rates *in vitro* (76), the small repeat motif sizes in our microsatellites suggest a low propensity to mutate in culture. Thus, our minimal set of five microsatellite markers should be robust in genotyping strains of diverse geographical and temporal origin. This approach can also be applied to multiplexed microsatellite genotyping of genetic cross progeny.

Taking the above assessments into account, we propose the following (Table 3): Parasite strain identity can be validated using PCR/Sanger sequencing-based SNP genotyping of parasite cultures, combined with microsatellite genotyping. These two methods could potentially be used to characterize parasite strains in large-scale surveys. Laboratory strain cross-contamination can be readily detected using FA microsatellite genotyping. This is especially useful when phenotyping P. falciparum genetic cross progeny, as it is critical to verify the identity and clonal purity of individual progeny to accurately map quantitative traits. To determine the multiplicity of infection in a field sample and identify new resistance determinants across the genome, more extensive methods such as amplicon sequencing and WGS are necessary (70, 85–89). Given the greater accessibility, ease, and throughput nature of our combined genotyping methods, we envision that these will be useful in the lab and field for routine use. Our adaptable genotyping methods can also help monitor the spread of novel drug-resistant traits once their causal determinants have been identified.

## MATERIALS AND METHODS

### Parasite cultures and strains.

Asexual blood stage P. falciparum parasites were cultured in human RBCs obtained from the Interstate Blood Bank (Memphis, TN) at 3% hematocrit, using RPMI 1640 supplemented with 25 mM HEPES, 2.1 g/liter sodium bicarbonate, 10 μg/ml gentamicin, 50 μM hypoxanthine, and 0.5% (wt/vol) AlbuMAX II (Thermo Fisher Scientific). Parasites were maintained at 37°C under 5% O_2_/5% CO_2_/90% N_2_ gas conditions. The GB4 parasite was obtained through BEI Resources, NIAID, NIH (MRA-925, contributed by Dr. Thomas Wellems). Other P. falciparum lines were kindly provided by Drs. Philip Rosenthal, Didier Ménard, Rick Fairhurst, Sarah Volkman, and Lise Musset (Table S5). Isolate 3D7 is a clone of NF54, originally isolated in 1979 in the Netherlands, and likely of African origin (41, 90).

### Simulated clinical samples.

NF54- or Cam3.II-infected packed RBCs were mixed with whole-blood obtained from the New York Blood Center and serially diluted 5-fold to generate four samples each with final parasitemias of 2.0% to 0.016% and hematocrits of 40%–50% (*N* = 2). Samples of 200 μl were used to make simulated dried blood spots (DBSs) on Whatman GB003 filter paper and processed 5 days later, while 100 μl each were used directly for simulated fresh whole-blood PCR, or frozen at−20°C and thawed 5 days later for simulated frozen whole-blood PCR.

### Ugandan DBSs.

We studied 16 isolates from patients who presented with symptomatic P. falciparum infections in eastern Uganda from April 2019 to June 2019, as described previously (43). In brief, subjects diagnosed with P. falciparum malaria and who consented to this study provided blood samples prior to malaria treatment. Blood was dried on Whatman 3MM filter paper, and samples were stored in zip-lock bags with desiccant at room temperature before extraction of DNA and molecular analysis. This study was approved by the Makerere University Research and Ethics Committee, the Uganda National Council of Science and Technology, and the University of California, San Francisco Committee on Human Research.

### SNP genotyping using PCR/Sanger sequencing.

The 40 μl PCRs consisted of 3 μl of 1%–5% parasitemia packed RBCs or simulated whole-blood samples, 0.125 μM final concentration of each forward and reverse primer, and 2× MyTaq Blood-PCR kit (Bioline) (supplemental methods). The following PCR conditions were used: 95°C for 3 min, 35 cycles: 95°C for 30 s, the appropriate annealing temperature and duration, and extension at 62°C for durations listed in Table S1. PCR products were submitted for Sanger sequencing (Genewiz) and analyzed with SeqMan Ultra (Version 17, DNASTAR). To generate NF54 and Cam3.II parasite mixtures, these lines were synchronized using 5% D-sorbitol (Sigma) and their parasitemias measured by flow cytometry (91) (*N* = 3). When starting with genomic DNA samples, the same annealing and extension PCR conditions were used, but the MyTaq polymerase was replaced with the 2× KAPA HiFi PCR Mix (Roche) in 15 μl total reaction volumes, and the denaturation step was performed at 98°C for 20 s. For each PCR reaction, we used 4 μl of gDNA from either Ugandan or simulated DBSs.

### Genomic DNA (gDNA) extraction.

gDNA was obtained from 3%–7% parasitemia cultures by lysing with 0.1% saponin in 1× PBS, and extracting with the QIAamp DNA Blood Midi Kit (Qiagen), with a combined RNase and Proteinase K treatment. DNA concentrations were determined using the Qubit dsDNA BR assay kit (Thermo Fisher Scientific). gDNA was extracted from both clinical (3 mm punches) and simulated DBSs (6 mm punches) using the QIAamp DNA Investigator kit (Qiagen) according to the manufacturer’s protocol, and eluted in 40 μl of AE buffer.

### Microsatellite PCR gel electrophoresis and FA genotyping.

PCRs with 15 μl volumes consisted of 12 ng of gDNA, 0.3 μM final concentration of each forward and reverse primer (with the forward primer fluorescently labeled for FA), and 2× KAPA HiFi PCR Mix (Roche) (supplemental methods). PCRs were run with the following conditions: 95°C for 3 min, 30 cycles: 98°C for 20 s, annealing temperature and duration as indicated in Table S2, and extension at 65°C for 15 s. For the gel electrophoresis method, 5 μl of each PCR product was run on a 2% agarose gel at 100V for 1.5 h–2 h with an Ultra Low Range DNA Ladder (Thermo Fisher Scientific) on both ends of the gel. The bands were visualized using a Gel Doc Imager (Bio-Rad) to identify the microsatellite sizes. For the FA method, fluorescently-labeled PCR products were treated with ExoSAP-IT Express Reagent (Thermo Fisher Scientific) and sent for capillary electrophoresis FA with the Liz500 ladder (Genewiz). Data were analyzed using Peak Scanner 2 (Thermo Fisher Scientific) to determine the microsatellite size of the fragment, which corresponds to the peak with the largest area in the electropherogram. The following parameters were used for analysis: peak sizes between 100 bp and 300 bp, peak height threshold of >500 RFU for the target channels (e.g., blue for 6-FAM) to eliminate PCR artifacts, and absolute and relative microsatellite sizes determined relative to the 3D7 reference strain. To multiplex FA, PCRs were set up individually for C2M18, BM5, C4M30, TA127, and C13M87, using forward primers fluorescently labeled with 6-FAM (blue), ATTO550/NED (yellow), or ATTO532/VIC (green) as listed in Table S2. PCR products from C2M18 and BM5 were mixed at a 2:1 ratio for each strain, and C4M30, TA127, and C13M87 were mixed at a 1:1:1 ratio, cleaned up with ExoSAP-IT, and sent for FA. Ugandan samples were run as 15 μl PCRs with 4 μl of gDNA for 35 cycles, cleaned up with EXO-SAP-IT, diluted 1:5, and sent for FA as singleplex reactions.

### Clustering of parasite strains.

The 12 laboratory strains and 16 Ugandan isolates (alongside two laboratory strain controls) were hierarchically clustered by average linkage using Gene Cluster 3.0 (92) and the resulting dendrograms were visualized using Java TreeView (93). For SNP clustering of laboratory strains, the alleles were input as follows: wild-type reference = 0; most common “major” alternate = 2; less common “minor” alternate = 1. For Ugandan samples, the alleles were input as: wild-type homozygous reference = 0; homozygous alternate = 2; and heterozygous = 1. For microsatellite clustering of both laboratory strains and Ugandan samples, the microsatellite sizes relative to 3D7 were used and only the major peaks were used for clustering the Ugandan isolates (Fig. 5; Tables 1, 2, and S4).

### Quantitative PCR (qPCR) assessment of *plasmepsin 2 (pm2)* copy number.

Multiplexed qPCR of *pm2* and single-copy *β-tubulin* (as an internal control) used TaqMan assays on a QuantStudio 3 real-time PCR system (Thermo Fisher Scientific), as described previously (26). Five standards of gene fragments, mixed at 1:1, 2:1, 3:1, 4:1, and 5:1 molar ratios of *pm2*:*β-tubulin*, were included as copy number controls (49). The reaction efficiency was calculated from previous runs, and the final copy number was calculated using the standard curve method for relative quantification (*n* = 3). Refer to the supplemental methods for the full protocol.

### Whole-genome sequencing (WGS) and microsatellite calling.

Parasite samples were subjected to WGS using the Illumina Nextera DNA Flex library preparation protocol. Briefly, 150 ng of gDNA was fragmented and tagmented using bead-linked transposons and subsequently amplified by five cycles of PCR to add dual-index adapter sequences to the DNA fragments. The libraries were quantified, pooled, and sequenced on Illumina MiSeq or NextSeq platforms. 3D7, NF54, GB4, 7G8, FCB, Cam3.II, RF12, RF7, Dd2, HB3, GC03, 1BB5, and 3BA6 were sequenced on a MiSeq instrument to generate paired-end 300 bp reads. F32, U659, and U815 were sequenced on a NextSeq 550 instrument to obtain 150 bp paired-end reads (Fig. S4C).

Sequence data were aligned to the P. falciparum 3D7 genome (PlasmoDB version 36.0) using Burrow-Wheeler Alignment (BWA). Reads that did not map to the reference genome and PCR duplicates were removed using SAMtools and Picard. The reads were realigned around indels using Genome Analyses Tool Kit (GATK) RealignerTargetCreator and base quality scores were recalibrated using GATK Table-Recalibration.

SAMTools mpileup was used to find SNPs in specified genomic regions (see Table 1) and were filtered based on quality scores (minimum base quality score ≥20, mapping quality >30, read depth ≥5) to obtain high-quality SNPs that were annotated with snpEFF. To determine the confidence of a microsatellite based on the number of reads, a custom Python script utilizing the pysam (94) module was written to call reads that harbored insertions or deletions at specified genomic loci within the respective windows (see Table 2). Integrative Genomics Viewer was used to verify the data.

## References

[1] WHO. 2020. World Malaria Report 2020. World Health Organization.

[2] Robson KJH, Walliker D, Creasey A, McBride J, Beale G, Wilson RJM. 1992. Cross-contamination of *Plasmodium* cultures. Parasitology Today 8:38–39. 10.1016/0169-4758(92)90075-D.

[3] Liu S, Mu J, Jiang H, Su XZ. 2008. Effects of *Plasmodium falciparum* mixed infections on *in vitro* antimalarial drug tests and genotyping. Am J Trop Med Hyg 79:178–184. 10.4269/ajtmh.2008.79.178. 18689621 PMC2680026

[4] Ranford-Cartwright LC, Balfe P, Carter R, Walliker D. 1991. Genetic hybrids of *Plasmodium falciparum* identified by amplification of genomic DNA from single oocysts. Mol Biochem Parasitol 49:239–243. 10.1016/0166-6851(91)90067-g. 1775167

[5] Felger I, Tavul L, Beck HP. 1993. *Plasmodium falciparum*: a rapid technique for genotyping the merozoite surface protein 2. Exp Parasitol 77:372–375. 10.1006/expr.1993.1094. 7901048

[6] Babiker H, Ranford-Cartwright L, Sultan A, Satti G, Walliker D. 1994. Genetic evidence that RI chloroquine resistance of *Plasmodium falciparum* is caused by recrudescence of resistant parasites. Trans R Soc Trop Med Hyg 88:328–331. 10.1016/0035-9203(94)90103-1. 7974680

[7] Paul RE, Packer MJ, Walmsley M, Lagog M, Ranford-Cartwright LC, Paru R, Day KP. 1995. Mating patterns in malaria parasite populations of Papua New Guinea. Science 269:1709–1711. 10.1126/science.7569897. 7569897

[8] Viriyakosol S, Siripoon N, Petcharapirat C, Petcharapirat P, Jarra W, Thaithong S, Brown KN, Snounou G. 1995. Genotyping of *Plasmodium falciparum* isolates by the polymerase chain reaction and potential uses in epidemiological studies. Bull World Health Organ 73:85–95. PMC2486592 7704931

[9] Daniels R, Volkman SK, Milner DA, Mahesh N, Neafsey DE, Park DJ, Rosen D, Angelino E, Sabeti PC, Wirth DF, Wiegand RC. 2008. A general SNP-based molecular barcode for *Plasmodium falciparum* identification and tracking. Malar J 7:223. 10.1186/1475-2875-7-223. 18959790 PMC2584654

[10] WHO. 2007. Methods and techniques for clinical trials on antimalarial drug efficacy: genotyping to identify parasite populations. World Health Organization.

[11] Felger I, Snounou G, Hastings I, Moehrle JJ, Beck HP. 2020. PCR correction strategies for malaria drug trials: updates and clarifications. Lancet Infect Dis 20:e20–e25. 10.1016/S1473-3099(19)30426-8. 31540841

[12] Gardner MJ, Hall N, Fung E, White O, Berriman M, Hyman RW, Carlton JM, Pain A, Nelson KE, Bowman S, Paulsen IT, James K, Eisen JA, Rutherford K, Salzberg SL, Craig A, Kyes S, Chan MS, Nene V, Shallom SJ, Suh B, Peterson J, Angiuoli S, Pertea M, Allen J, Selengut J, Haft D, Mather MW, Vaidya AB, Martin DM, Fairlamb AH, Fraunholz MJ, Roos DS, Ralph SA, McFadden GI, Cummings LM, Subramanian GM, Mungall C, Venter JC, Carucci DJ, Hoffman SL, Newbold C, Davis RW, Fraser CM, Barrell B. 2002. Genome sequence of the human malaria parasite *Plasmodium falciparum*. Nature 419:498–511. 10.1038/nature01097. 12368864 PMC3836256

[13] Kunkel TA. 1986. Frameshift mutagenesis by eucaryotic DNA polymerases *in vitro*. J Biol Chem 261:13581–13587. 10.1016/S0021-9258(18)67059-0. 3759982

[14] Ferdig MT, Su XZ. 2000. Microsatellite markers and genetic mapping in *Plasmodium falciparum*. Parasitol Today 16:307–312. 10.1016/s0169-4758(00)01676-8. 10858651

[15] Blasco B, Leroy D, Fidock DA. 2017. Antimalarial drug resistance: linking *Plasmodium falciparum* parasite biology to the clinic. Nat Med 23:917–928. 10.1038/nm.4381. 28777791 PMC5747363

[16] Volkman SK, Herman J, Lukens AK, Hartl DL. 2017. Genome-wide association studies of drug-resistance determinants. Trends Parasitol 33:214–230. 10.1016/j.pt.2016.10.001. 28179098

[17] Apinjoh TO, Ouattara A, Titanji VPK, Djimde A, Amambua-Ngwa A. 2019. Genetic diversity and drug resistance surveillance of *Plasmodium falciparum* for malaria elimination: is there an ideal tool for resource-limited sub-Saharan Africa? Malar J 18:217. 10.1186/s12936-019-2844-5. 31242921 PMC6595576

[18] Okombo J, Kanai M, Deni I, Fidock DA. 2021. Genomic and genetic approaches to studying antimalarial drug resistance and *Plasmodium* biology. Trends Parasitol 37:476–492. 10.1016/j.pt.2021.02.007. 33715941 PMC8162148

[19] Reed MB, Saliba KJ, Caruana SR, Kirk K, Cowman AF. 2000. Pgh1 modulates sensitivity and resistance to multiple antimalarials in *Plasmodium falciparum*. Nature 403:906–909. 10.1038/35002615. 10706290

[20] Cooper RA, Ferdig MT, Su XZ, Ursos LM, Mu J, Nomura T, Fujioka H, Fidock DA, Roepe PD, Wellems TE. 2002. Alternative mutations at position 76 of the vacuolar transmembrane protein PfCRT are associated with chloroquine resistance and unique stereospecific quinine and quinidine responses in *Plasmodium falciparum*. Mol Pharmacol 61:35–42. 10.1124/mol.61.1.35. 11752204

[21] Mu J, Ferdig MT, Feng X, Joy DA, Duan J, Furuya T, Subramanian G, Aravind L, Cooper RA, Wootton JC, Xiong M, Su XZ. 2003. Multiple transporters associated with malaria parasite responses to chloroquine and quinine. Mol Microbiol 49:977–989. 10.1046/j.1365-2958.2003.03627.x. 12890022

[22] Fidock DA, Nomura T, Talley AK, Cooper RA, Dzekunov SM, Ferdig MT, Ursos LM, Sidhu AB, Naude B, Deitsch KW, Su XZ, Wootton JC, Roepe PD, Wellems TE. 2000. Mutations in the *P. falciparum* digestive vacuole transmembrane protein PfCRT and evidence for their role in chloroquine resistance. Mol Cell 6:861–871. 10.1016/S1097-2765(05)00077-8. 11090624 PMC2944663

[23] Sidhu AB, Verdier-Pinard D, Fidock DA. 2002. Chloroquine resistance in *Plasmodium falciparum* malaria parasites conferred by *pfcrt* mutations. Science 298:210–213. 10.1126/science.1074045. 12364805 PMC2954758

[24] Veiga MI, Dhingra SK, Henrich PP, Straimer J, Gnadig N, Uhlemann AC, Martin RE, Lehane AM, Fidock DA. 2016. Globally prevalent PfMDR1 mutations modulate *Plasmodium falciparum* susceptibility to artemisinin-based combination therapies. Nat Commun 7:11553. 10.1038/ncomms11553. 27189525 PMC4873939

[25] Bopp S, Magistrado P, Wong W, Schaffner SF, Mukherjee A, Lim P, Dhorda M, Amaratunga C, Woodrow CJ, Ashley EA, White NJ, Dondorp AM, Fairhurst RM, Ariey F, Menard D, Wirth DF, Volkman SK. 2018. *Plasmepsin II-III* copy number accounts for bimodal piperaquine resistance among Cambodian *Plasmodium falciparum*. Nat Commun 9:1769. 10.1038/s41467-018-04104-z. 29720620 PMC5931971

[26] Ross LS, Dhingra SK, Mok S, Yeo T, Wicht KJ, Kumpornsin K, Takala-Harrison S, Witkowski B, Fairhurst RM, Ariey F, Menard D, Fidock DA. 2018. Emerging Southeast Asian PfCRT mutations confer *Plasmodium falciparum* resistance to the first-line antimalarial piperaquine. Nat Commun 9:3314. 10.1038/s41467-018-05652-0. 30115924 PMC6095916

[27] Dhingra SK, Small-Saunders JL, Menard D, Fidock DA. 2019. *Plasmodium falciparum* resistance to piperaquine driven by PfCRT. Lancet Infect Dis 19:1168–1169. 10.1016/S1473-3099(19)30543-2. 31657776 PMC6943240

[28] Humphreys GS, Merinopoulos I, Ahmed J, Whitty CJ, Mutabingwa TK, Sutherland CJ, Hallett RL. 2007. Amodiaquine and artemether-lumefantrine select distinct alleles of the *Plasmodium falciparum mdr1* gene in Tanzanian children treated for uncomplicated malaria. Antimicrob Agents Chemother 51:991–997. 10.1128/AAC.00875-06. 17194834 PMC1803116

[29] Sa JM, Twu O, Hayton K, Reyes S, Fay MP, Ringwald P, Wellems TE. 2009. Geographic patterns of *Plasmodium falciparum* drug resistance distinguished by differential responses to amodiaquine and chloroquine. Proc Natl Acad Sci USA 106:18883–18889. 10.1073/pnas.0911317106. 19884511 PMC2771746

[30] Beshir K, Sutherland CJ, Merinopoulos I, Durrani N, Leslie T, Rowland M, Hallett RL. 2010. Amodiaquine resistance in *Plasmodium falciparum* malaria in Afghanistan is associated with the *pfcrt* SVMNT allele at codons 72 to 76. Antimicrob Agents Chemother 54:3714–3716. 10.1128/AAC.00358-10. 20547800 PMC2934991

[31] Venkatesan M, Gadalla NB, Stepniewska K, Dahal P, Nsanzabana C, Moriera C, Price RN, Martensson A, Rosenthal PJ, Dorsey G, Sutherland CJ, Guerin P, Davis TME, Menard D, Adam I, Ademowo G, Arze C, Baliraine FN, Berens-Riha N, Bjorkman A, Borrmann S, Checchi F, Desai M, Dhorda M, Djimde AA, El-Sayed BB, Eshetu T, Eyase F, Falade C, Faucher JF, Froberg G, Grivoyannis A, Hamour S, Houze S, Johnson J, Kamugisha E, Kariuki S, Kiechel JR, Kironde F, Kofoed PE, LeBras J, Malmberg M, Mwai L, Ngasala B, Nosten F, Nsobya SL, Nzila A, Oguike M, Otienoburu SD, Ogutu B, ASAQ Molecular Marker Study Group, et al. 2014. Polymorphisms in *Plasmodium falciparum* chloroquine resistance transporter and multidrug resistance 1 genes: parasite risk factors that affect treatment outcomes for *P falciparum* malaria after artemether-lumefantrine and artesunate-amodiaquine. Am. J Trop Med Hyg 91:833–843. 10.4269/ajtmh.14-0031. PMC4183414 25048375

[32] Preechapornkul P, Imwong M, Chotivanich K, Pongtavornpinyo W, Dondorp AM, Day NP, White NJ, Pukrittayakamee S. 2009. *Plasmodium falciparum pfmdr1* amplification, mefloquine resistance, and parasite fitness. Antimicrob Agents Chemother 53:1509–1515. 10.1128/AAC.00241-08. 19164150 PMC2663078

[33] Phyo AP, Ashley EA, Anderson TJC, Bozdech Z, Carrara VI, Sriprawat K, Nair S, White MM, Dziekan J, Ling C, Proux S, Konghahong K, Jeeyapant A, Woodrow CJ, Imwong M, McGready R, Lwin KM, Day NPJ, White NJ, Nosten F. 2016. Declining efficacy of artemisinin combination therapy against *P. falciparum* malaria on the Thai-Myanmar border (2003–2013): the role of parasite genetic factors. Clin Infect Dis 63:784–791. 10.1093/cid/ciw388. 27313266 PMC4996140

[34] Gregson A, Plowe CV. 2005. Mechanisms of resistance of malaria parasites to antifolates. Pharmacol Rev 57:117–145. 10.1124/pr.57.1.4. 15734729

[35] Okell LC, Griffin JT, Roper C. 2017. Mapping sulphadoxine-pyrimethamine-resistant *Plasmodium falciparum* malaria in infected humans and in parasite populations in Africa. Sci Rep 7:7389. 10.1038/s41598-017-06708-9. 28785011 PMC5547055

[36] Ariey F, Witkowski B, Amaratunga C, Beghain J, Langlois AC, Khim N, Kim S, Duru V, Bouchier C, Ma L, Lim P, Leang R, Duong S, Sreng S, Suon S, Chuor CM, Bout DM, Menard S, Rogers WO, Genton B, Fandeur T, Miotto O, Ringwald P, Le Bras J, Berry A, Barale JC, Fairhurst RM, Benoit-Vical F, Mercereau-Puijalon O, Menard D. 2014. A molecular marker of artemisinin-resistant *Plasmodium falciparum* malaria. Nature 505:50–55. 10.1038/nature12876. 24352242 PMC5007947

[37] Straimer J, Gnadig NF, Witkowski B, Amaratunga C, Duru V, Ramadani AP, Dacheux M, Khim N, Zhang L, Lam S, Gregory PD, Urnov FD, Mercereau-Puijalon O, Benoit-Vical F, Fairhurst RM, Menard D, Fidock DA. 2015. K13-propeller mutations confer artemisinin resistance in *Plasmodium falciparum* clinical isolates. Science 347:428–431. 10.1126/science.1260867. 25502314 PMC4349400

[38] Imwong M, Dhorda M, Myo Tun K, Thu AM, Phyo AP, Proux S, Suwannasin K, Kunasol C, Srisutham S, Duanguppama J, Vongpromek R, Promnarate C, Saejeng A, Khantikul N, Sugaram R, Thanapongpichat S, Sawangjaroen N, Sutawong K, Han KT, Htut Y, Linn K, Win AA, Hlaing TM, van der Pluijm RW, Mayxay M, Pongvongsa T, Phommasone K, Tripura R, Peto TJ, von Seidlein L, Nguon C, Lek D, Chan XHS, Rekol H, Leang R, Huch C, Kwiatkowski DP, Miotto O, Ashley EA, Kyaw MP, Pukrittayakamee S, Day NPJ, Dondorp AM, Smithuis FM, Nosten FH, White NJ. 2020. Molecular epidemiology of resistance to antimalarial drugs in the Greater Mekong subregion: an observational study. Lancet Infect Dis 20:1470–1480. 10.1016/S1473-3099(20)30228-0. 32679084 PMC7689289

[39] Mathieu LC, Cox H, Early AM, Mok S, Lazrek Y, Paquet JC, Ade MP, Lucchi NW, Grant Q, Udhayakumar V, Alexandre JS, Demar M, Ringwald P, Neafsey DE, Fidock DA, Musset L. 2020. Local emergence in Amazonia of *Plasmodium falciparum k13* C580Y mutants associated with *in vitro* artemisinin resistance. Elife 9:e51015. 10.7554/eLife.51015. 32394893 PMC7217694

[40] Miotto O, Sekihara M, Tachibana SI, Yamauchi M, Pearson RD, Amato R, Goncalves S, Mehra S, Noviyanti R, Marfurt J, Auburn S, Price RN, Mueller I, Ikeda M, Mori T, Hirai M, Tavul L, Hetzel MW, Laman M, Barry AE, Ringwald P, Ohashi J, Hombhanje F, Kwiatkowski DP, Mita T. 2020. Emergence of artemisinin-resistant *Plasmodium falciparum* with *kelch13* C580Y mutations on the island of New Guinea. PLoS Pathog 16:e1009133. 10.1371/journal.ppat.1009133. 33320907 PMC7771869

[41] Uwimana A, Legrand E, Stokes BH, Ndikumana JM, Warsame M, Umulisa N, Ngamije D, Munyaneza T, Mazarati JB, Munguti K, Campagne P, Criscuolo A, Ariey F, Murindahabi M, Ringwald P, Fidock DA, Mbituyumuremyi A, Menard D. 2020. Emergence and clonal expansion of *in vitro* artemisinin-resistant *Plasmodium falciparum kelch13* R561H mutant parasites in Rwanda. Nat Med 26:1602–1608. 10.1038/s41591-020-1005-2. 32747827 PMC7541349

[42] Stokes BH, Dhingra SK, Rubiano K, Mok S, Straimer J, Gnadig NF, Deni I, Schindler KA, Bath JR, Ward KE, Striepen J, Yeo T, Ross LS, Legrand E, Ariey F, Cunningham CH, Souleymane IM, Gansane A, Nzoumbou-Boko R, Ndayikunda C, Kabanywanyi AM, Uwimana A, Smith SJ, Kolley O, Ndounga M, Warsame M, Leang R, Nosten F, Anderson TJ, Rosenthal PJ, Menard D, Fidock DA. 2021. *Plasmodium falciparum* K13 mutations in Africa and Asia impact artemisinin resistance and parasite fitness. Elife 10:e66277. 10.7554/eLife.66277. 34279219 PMC8321553

[43] Asua V, Conrad MD, Aydemir O, Duvalsaint M, Legac J, Duarte E, Tumwebaze P, Chin DM, Cooper RA, Yeka A, Kamya MR, Dorsey G, Nsobya SL, Bailey J, Rosenthal PJ. 2021. Changing pevalence of potential mediators of aminoquinoline, antifolate, and artemisinin resistance across Uganda. J Infect Dis 223:985–994. 10.1093/infdis/jiaa687. 33146722 PMC8006419

[44] Uwimana A, Umulisa N, Venkatesan M, Svigel SS, Zhou Z, Munyaneza T, Habimana RM, Rucogoza A, Moriarty LF, Sandford R, Piercefield E, Goldman I, Ezema B, Talundzic E, Pacheco MA, Escalante AA, Ngamije D, Mangala JN, Kabera M, Munguti K, Murindahabi M, Brieger W, Musanabaganwa C, Mutesa L, Udhayakumar V, Mbituyumuremyi A, Halsey ES, Lucchi NW. 2021. Association of *Plasmodium falciparum* *kelch13* R561H genotypes with delayed parasite clearance in Rwanda: an open-label, single-arm, multicentre, therapeutic efficacy study. Lancet Infect Dis 21:1120–1128. 10.1016/S1473-3099(21)00142-0. 33864801 PMC10202849

[45] Rosenthal PJ. 2021. Are artemisinin-based combination therapies for malaria beginning to fail in Africa? Am J Trop Med Hyg Epub ahead of print. 10.4269/ajtmh.21-0797. PMC8592154 34491214

[46] Wootton JC, Feng X, Ferdig MT, Cooper RA, Mu J, Baruch DI, Magill AJ, Su XZ. 2002. Genetic diversity and chloroquine selective sweeps in *Plasmodium falciparum*. Nature 418:320–323. 10.1038/nature00813. 12124623

[47] Imwong M, Suwannasin K, Kunasol C, Sutawong K, Mayxay M, Rekol H, Smithuis FM, Hlaing TM, Tun KM, van der Pluijm RW, Tripura R, Miotto O, Menard D, Dhorda M, Day NPJ, White NJ, Dondorp AM. 2017. The spread of artemisinin-resistant *Plasmodium falciparum* in the Greater Mekong subregion: a molecular epidemiology observational study. Lancet Infect Dis 17:491–497. 10.1016/S1473-3099(17)30048-8. 28161569 PMC5406483

[48] Amaratunga C, Lim P, Suon S, Sreng S, Mao S, Sopha C, Sam B, Dek D, Try V, Amato R, Blessborn D, Song L, Tullo GS, Fay MP, Anderson JM, Tarning J, Fairhurst RM. 2016. Dihydroartemisinin-piperaquine resistance in *Plasmodium falciparum* malaria in Cambodia: a multisite prospective cohort study. Lancet Infect Dis 16:357–365. 10.1016/S1473-3099(15)00487-9. 26774243 PMC4792715

[49] Witkowski B, Duru V, Khim N, Ross LS, Saintpierre B, Beghain J, Chy S, Kim S, Ke S, Kloeung N, Eam R, Khean C, Ken M, Loch K, Bouillon A, Domergue A, Ma L, Bouchier C, Leang R, Huy R, Nuel G, Barale JC, Legrand E, Ringwald P, Fidock DA, Mercereau-Puijalon O, Ariey F, Menard D. 2017. A surrogate marker of piperaquine-resistant *Plasmodium falciparum* malaria: a phenotype-genotype association study. Lancet Infect Dis 17:174–183. 10.1016/S1473-3099(16)30415-7. 27818097 PMC5266792

[50] Amato R, Lim P, Miotto O, Amaratunga C, Dek D, Pearson RD, Almagro-Garcia J, Neal AT, Sreng S, Suon S, Drury E, Jyothi D, Stalker J, Kwiatkowski DP, Fairhurst RM. 2017. Genetic markers associated with dihydroartemisinin-piperaquine failure in *Plasmodium falciparum* malaria in Cambodia: A genotype-phenotype association study. Lancet Infect Dis 17:164–173. 10.1016/S1473-3099(16)30409-1. 27818095 PMC5564489

[51] Hamilton WL, Amato R, van der Pluijm RW, Jacob CG, Quang HH, Thuy-Nhien NT, Hien TT, Hongvanthong B, Chindavongsa K, Mayxay M, Huy R, Leang R, Huch C, Dysoley L, Amaratunga C, Suon S, Fairhurst RM, Tripura R, Peto TJ, Sovann Y, Jittamala P, Hanboonkunupakarn B, Pukrittayakamee S, Chau NH, Imwong M, Dhorda M, Vongpromek R, Chan XHS, Maude RJ, Pearson RD, Nguyen T, Rockett K, Drury E, Goncalves S, White NJ, Day NP, Kwiatkowski DP, Dondorp AM, Miotto O. 2019. Evolution and expansion of multidrug-resistant malaria in southeast Asia: a genomic epidemiology study. Lancet Infect Dis 19:943–951. 10.1016/S1473-3099(19)30392-5. 31345709 PMC6715858

[52] van der Pluijm RW, Imwong M, Chau NH, Hoa NT, Thuy-Nhien NT, Thanh NV, Jittamala P, Hanboonkunupakarn B, Chutasmit K, Saelow C, Runjarern R, Kaewmok W, Tripura R, Peto TJ, Yok S, Suon S, Sreng S, Mao S, Oun S, Yen S, Amaratunga C, Lek D, Huy R, Dhorda M, Chotivanich K, Ashley EA, Mukaka M, Waithira N, Cheah PY, Maude RJ, Amato R, Pearson RD, Goncalves S, Jacob CG, Hamilton WL, Fairhurst RM, Tarning J, Winterberg M, Kwiatkowski DP, Pukrittayakamee S, Hien TT, Day NP, Miotto O, White NJ, Dondorp AM. 2019. Determinants of dihydroartemisinin-piperaquine treatment failure in *Plasmodium falciparum* malaria in Cambodia, Thailand, and Vietnam: a prospective clinical, pharmacological, and genetic study. Lancet Infect Dis 19:952–961. 10.1016/S1473-3099(19)30391-3. 31345710 PMC6715822

[53] Imwong M, Suwannasin K, Srisutham S, Vongpromek R, Promnarate C, Saejeng A, Phyo AP, Proux S, Pongvongsa T, Nguon C, Miotto O, Tripura R, Chau NH, Lek D, Dang Trung Nghia H, Peto TJ, Callery JJ, van der Pluijm RW, Amaratunga C, Mukaka M, von Seidlein L, Mayxay M, Thuy-Nhien NT, Newton PN, Day NP, Ashley EA, Nosten FH, Smithuis FM, Dhorda M, White NJ, Dondorp AM. 2021. Evolution of multidrug resistance in *Plasmodium falciparum*: a longitudinal study of genetic resistance markers in the Greater Mekong Subregion. Antimicrob Agents Chemother Epub ahead of print. 10.1128/AAC.01121-21. PMC8597770 34516247

[54] Su X, Wellems TE. 1996. Toward a high-resolution *Plasmodium falciparum* linkage map: polymorphic markers from hundreds of simple sequence repeats. Genomics 33:430–444. 10.1006/geno.1996.0218. 8661002

[55] Anderson TJ, Su XZ, Bockarie M, Lagog M, Day KP. 1999. Twelve microsatellite markers for characterization of *Plasmodium falciparum* from finger-prick blood samples. Parasitology 119:113–125. 10.1017/S0031182099004552. 10466118

[56] Figan CE, Sa JM, Mu J, Melendez-Muniz VA, Liu CH, Wellems TE. 2018. A set of microsatellite markers to differentiate *Plasmodium falciparum* progeny of four genetic crosses. Malar J 17:60. 10.1186/s12936-018-2210-z. 29394891 PMC5797376

[57] Oyola SO, Otto TD, Gu Y, Maslen G, Manske M, Campino S, Turner DJ, Macinnis B, Kwiatkowski DP, Swerdlow HP, Quail MA. 2012. Optimizing Illumina next-generation sequencing library preparation for extremely AT-biased genomes. BMC Genomics 13:1. 10.1186/1471-2164-13-1. 22214261 PMC3312816

[58] Vendrely KM, Kumar S, Li X, Vaughan AM. 2020. Humanized mice and the rebirth of malaria genetic crosses. Trends Parasitol 36:850–863. 10.1016/j.pt.2020.07.009. 32891493 PMC7530086

[59] Wellems TE, Panton LJ, Gluzman IY, do Rosario VE, Gwadz RW, Walker-Jonah A, Krogstad DJ. 1990. Chloroquine resistance not linked to *mdr*-like genes in a *Plasmodium falciparum* cross. Nature 345:253–255. 10.1038/345253a0. 1970614

[60] Amato R, Pearson RD, Almagro-Garcia J, Amaratunga C, Lim P, Suon S, Sreng S, Drury E, Stalker J, Miotto O, Fairhurst RM, Kwiatkowski DP. 2018. Origins of the current outbreak of multidrug-resistant malaria in southeast Asia: a retrospective genetic study. Lancet Infect Dis 18:337–345. 10.1016/S1473-3099(18)30068-9. 29398391 PMC5835763

[61] Neafsey DE, Volkman SK. 2017. Malaria genomics in the era of eradication. Cold Spring Harb Perspect Med 7:a025544. 10.1101/cshperspect.a025544. 28389516 PMC5538406

[62] Amambua-Ngwa A, Amenga-Etego L, Kamau E, Amato R, Ghansah A, Golassa L, Randrianarivelojosia M, Ishengoma D, Apinjoh T, Maiga-Ascofare O, Andagalu B, Yavo W, Bouyou-Akotet M, Kolapo O, Mane K, Worwui A, Jeffries D, Simpson V, D'Alessandro U, Kwiatkowski D, Djimde AA. 2019. Major subpopulations of *Plasmodium falciparum* in sub-Saharan Africa. Science 365:813–816. 10.1126/science.aav5427. 31439796

[63] Campino S, Auburn S, Kivinen K, Zongo I, Ouedraogo JB, Mangano V, Djimde A, Doumbo OK, Kiara SM, Nzila A, Borrmann S, Marsh K, Michon P, Mueller I, Siba P, Jiang H, Su XZ, Amaratunga C, Socheat D, Fairhurst RM, Imwong M, Anderson T, Nosten F, White NJ, Gwilliam R, Deloukas P, MacInnis B, Newbold CI, Rockett K, Clark TG, Kwiatkowski DP. 2011. Population genetic analysis of *Plasmodium falciparum* parasites using a customized Illumina GoldenGate genotyping assay. PLoS One 6:e20251. 10.1371/journal.pone.0020251. 21673999 PMC3108946

[64] Phyo AP, Nkhoma S, Stepniewska K, Ashley EA, Nair S, McGready R, Ler Moo C, Al-Saai S, Dondorp AM, Lwin KM, Singhasivanon P, Day NP, White NJ, Anderson TJ, Nosten F. 2012. Emergence of artemisinin-resistant malaria on the western border of Thailand: a longitudinal study. Lancet 379:1960–1966. 10.1016/S0140-6736(12)60484-X. 22484134 PMC3525980

[65] Preston MD, Campino S, Assefa SA, Echeverry DF, Ocholla H, Amambua-Ngwa A, Stewart LB, Conway DJ, Borrmann S, Michon P, Zongo I, Ouedraogo JB, Djimde AA, Doumbo OK, Nosten F, Pain A, Bousema T, Drakeley CJ, Fairhurst RM, Sutherland CJ, Roper C, Clark TG. 2014. A barcode of organellar genome polymorphisms identifies the geographic origin of *Plasmodium falciparum* strains. Nat Commun 5:4052. 10.1038/ncomms5052. 24923250 PMC4082634

[66] Pholwat S, Liu J, Stroup S, Jacob ST, Banura P, Moore CC, Huang F, Laufer MK, Houpt E, Guler JL. 2017. The malaria TaqMan array card includes 87 assays for *Plasmodium falciparum* drug resistance, identification of species, and genotyping in a single reaction. Antimicrob Agents Chemother 61:e00110-17. 10.1128/AAC.00110-17. 28264857 PMC5404514

[67] Leelawong M, Adams NM, Gabella WE, Wright DW, Haselton FR. 2019. Detection of single-nucleotide polymorphism markers of antimalarial drug resistance directly from whole blood. J Mol Diagn 21:623–631. 10.1016/j.jmoldx.2019.02.004. 31204166 PMC6650786

[68] Daniels R, Ndiaye D, Wall M, McKinney J, Sene PD, Sabeti PC, Volkman SK, Mboup S, Wirth DF. 2012. Rapid, field-deployable method for genotyping and discovery of single-nucleotide polymorphisms associated with drug resistance in *Plasmodium falciparum*. Antimicrob Agents Chemother 56:2976–2986. 10.1128/AAC.05737-11. 22430961 PMC3370755

[69] Baniecki ML, Faust AL, Schaffner SF, Park DJ, Galinsky K, Daniels RF, Hamilton E, Ferreira MU, Karunaweera ND, Serre D, Zimmerman PA, Sa JM, Wellems TE, Musset L, Legrand E, Melnikov A, Neafsey DE, Volkman SK, Wirth DF, Sabeti PC. 2015. Development of a single nucleotide polymorphism barcode to genotype *Plasmodium vivax* infections. PLoS Negl Trop Dis 9:e0003539. 10.1371/journal.pntd.0003539. 25781890 PMC4362761

[70] Lerch A, Koepfli C, Hofmann NE, Messerli C, Wilcox S, Kattenberg JH, Betuela I, O'Connor L, Mueller I, Felger I. 2017. Development of amplicon deep sequencing markers and data analysis pipeline for genotyping multi-clonal malaria infections. BMC Genomics 18:864. 10.1186/s12864-017-4260-y. 29132317 PMC5682641

[71] Jacob CG, Thuy-Nhien N, Mayxay M, Maude RJ, Quang HH, Hongvanthong B, Vanisaveth V, Ngo Duc T, Rekol H, van der Pluijm R, von Seidlein L, Fairhurst R, Nosten F, Hossain MA, Park N, Goodwin S, Ringwald P, Chindavongsa K, Newton P, Ashley E, Phalivong S, Maude R, Leang R, Huch C, Dong LT, Nguyen KT, Nhat TM, Hien TT, Nguyen H, Zdrojewski N, Canavati S, Sayeed AA, Uddin D, Buckee C, Fanello CI, Onyamboko M, Peto T, Tripura R, Amaratunga C, Myint Thu A, Delmas G, Landier J, Parker DM, Chau NH, Lek D, Suon S, Callery J, Jittamala P, Hanboonkunupakarn B, Pukrittayakamee S, et al. 2021. Genetic surveillance in the Greater Mekong subregion and South Asia to support malaria control and elimination. Elife 10:e62997. 10.7554/eLife.62997. 34372970 PMC8354633

[72] Aydemir O, Janko M, Hathaway NJ, Verity R, Mwandagalirwa MK, Tshefu AK, Tessema SK, Marsh PW, Tran A, Reimonn T, Ghani AC, Ghansah A, Juliano JJ, Greenhouse BR, Emch M, Meshnick SR, Bailey JA. 2018. Drug-resistance and population structure of *Plasmodium falciparum* across the Democratic Republic of Congo using high-throughput molecular inversion probes. J Infect Dis 218:946–955. 10.1093/infdis/jiy223. 29718283 PMC6093412

[73] Tirrell AR, Vendrely KM, Checkley LA, Davis SZ, McDew-White M, Cheeseman IH, Vaughan AM, Nosten FH, Anderson TJC, Ferdig MT. 2019. Pairwise growth competitions identify relative fitness relationships among artemisinin resistant *Plasmodium falciparum* field isolates. Malar J 18:295. 10.1186/s12936-019-2934-4. 31462253 PMC6714446

[74] Salipante SJ, Scroggins SM, Hampel HL, Turner EH, Pritchard CC. 2014. Microsatellite instability detection by next generation sequencing. Clin Chem 60:1192–1199. 10.1373/clinchem.2014.223677. 24987110

[75] Baudrin LG, Deleuze JF, How-Kit A. 2018. Molecular and computational methods for the detection of microsatellite instability in cancer. Front Oncol 8:621. 10.3389/fonc.2018.00621. 30631754 PMC6315116

[76] McDew-White M, Li X, Nkhoma SC, Nair S, Cheeseman I, Anderson TJC. 2019. Mode and tempo of microsatellite length change in a malaria parasite mutation accumulation experiment. Genome Biol Evol 11:1971–1985. 10.1093/gbe/evz140. 31273388 PMC6644851

[77] Lander ES, Linton LM, Birren B, Nusbaum C, Zody MC, Baldwin J, Devon K, Dewar K, Doyle M, FitzHugh W, Funke R, Gage D, Harris K, Heaford A, Howland J, Kann L, Lehoczky J, LeVine R, McEwan P, McKernan K, Meldrim J, Mesirov JP, Miranda C, Morris W, Naylor J, Raymond C, Rosetti M, Santos R, Sheridan A, Sougnez C, Stange-Thomann Y, Stojanovic N, Subramanian A, Wyman D, Rogers J, Sulston J, Ainscough R, Beck S, Bentley D, Burton J, Clee C, Carter N, Coulson A, Deadman R, Deloukas P, Dunham A, Dunham I, Durbin R, French L, Grafham D, International Human Genome Sequencing Consortium, et al. 2001. Initial sequencing and analysis of the human genome. Nature 409:860–921. 10.1038/35057062. 11237011

[78] Miles A, Iqbal Z, Vauterin P, Pearson R, Campino S, Theron M, Gould K, Mead D, Drury E, O'Brien J, Ruano Rubio V, MacInnis B, Mwangi J, Samarakoon U, Ranford-Cartwright L, Ferdig M, Hayton K, Su XZ, Wellems T, Rayner J, McVean G, Kwiatkowski D. 2016. Indels, structural variation, and recombination drive genomic diversity in *Plasmodium falciparum*. Genome Res 26:1288–1299. 10.1101/gr.203711.115. 27531718 PMC5052046

[79] Weber JL, Wong C. 1993. Mutation of human short tandem repeats. Hum Mol Genet 2:1123–1128. 10.1093/hmg/2.8.1123. 8401493

[80] Bhargava A, Fuentes FF. 2010. Mutational dynamics of microsatellites. Mol Biotechnol 44:250–266. 10.1007/s12033-009-9230-4. 20012711

[81] Lee AH, Symington LS, Fidock DA. 2014. DNA repair mechanisms and their biological roles in the malaria parasite *Plasmodium falciparum*. Microbiol Mol Biol Rev 78:469–486. 10.1128/MMBR.00059-13. 25184562 PMC4187680

[82] Kirkman LA, Lawrence EA, Deitsch KW. 2014. Malaria parasites utilize both homologous recombination and alternative end joining pathways to maintain genome integrity. Nucleic Acids Res 42:370–379. 10.1093/nar/gkt881. 24089143 PMC3874194

[83] Su X, Ferdig MT, Huang Y, Huynh CQ, Liu A, You J, Wootton JC, Wellems TE. 1999. A genetic map and recombination parameters of the human malaria parasite *Plasmodium falciparum*. Science 286:1351–1353. 10.1126/science.286.5443.1351. 10558988

[84] Zhang M, Wang C, Otto TD, Oberstaller J, Liao X, Adapa SR, Udenze K, Bronner IF, Casandra D, Mayho M, Brown J, Li S, Swanson J, Rayner JC, Jiang RHY, Adams JH. 2018. Uncovering the essential genes of the human malaria parasite *Plasmodium falciparum* by saturation mutagenesis. Science 360:eaap7847. 10.1126/science.aap7847. 29724925 PMC6360947

[85] Robinson T, Campino SG, Auburn S, Assefa SA, Polley SD, Manske M, MacInnis B, Rockett KA, Maslen GL, Sanders M, Quail MA, Chiodini PL, Kwiatkowski DP, Clark TG, Sutherland CJ. 2011. Drug-resistant genotypes and multi-clonality in *Plasmodium falciparum* analysed by direct genome sequencing from peripheral blood of malaria patients. PLoS One 6:e23204. 10.1371/journal.pone.0023204. 21853089 PMC3154926

[86] Manske M, Miotto O, Campino S, Auburn S, Almagro-Garcia J, Maslen G, O'Brien J, Djimde A, Doumbo O, Zongo I, Ouedraogo JB, Michon P, Mueller I, Siba P, Nzila A, Borrmann S, Kiara SM, Marsh K, Jiang H, Su XZ, Amaratunga C, Fairhurst R, Socheat D, Nosten F, Imwong M, White NJ, Sanders M, Anastasi E, Alcock D, Drury E, Oyola S, Quail MA, Turner DJ, Ruano-Rubio V, Jyothi D, Amenga-Etego L, Hubbart C, Jeffreys A, Rowlands K, Sutherland C, Roper C, Mangano V, Modiano D, Tan JC, Ferdig MT, Amambua-Ngwa A, Conway DJ, Takala-Harrison S, Plowe CV, Rayner JC, et al. 2012. Analysis of *Plasmodium falciparum* diversity in natural infections by deep sequencing. Nature 487:375–379. 10.1038/nature11174. 22722859 PMC3738909

[87] Wong W, Griggs AD, Daniels RF, Schaffner SF, Ndiaye D, Bei AK, Deme AB, MacInnis B, Volkman SK, Hartl DL, Neafsey DE, Wirth DF. 2017. Genetic relatedness analysis reveals the cotransmission of genetically related *Plasmodium falciparum* parasites in Thies, Senegal. Genome Med 9:5. 10.1186/s13073-017-0398-0. 28118860 PMC5260019

[88] Early AM, Daniels RF, Farrell TM, Grimsby J, Volkman SK, Wirth DF, MacInnis BL, Neafsey DE. 2019. Detection of low-density *Plasmodium falciparum* infections using amplicon deep sequencing. Malar J 18:219. 10.1186/s12936-019-2856-1. 31262308 PMC6604269

[89] Yang T, Ottilie S, Istvan ES, Godinez-Macias KP, Lukens AK, Baragana B, Campo B, Walpole C, Niles JC, Chibale K, Dechering KJ, Llinas M, Lee MCS, Kato N, Wyllie S, McNamara CW, Gamo FJ, Burrows J, Fidock DA, Goldberg DE, Gilbert IH, Wirth DF, Winzeler EA, the Malaria Drug Accelerator Consortium. 2021. MalDA, accelerating malaria drug discovery. Trends Parasitol 37:493–507. 10.1016/j.pt.2021.01.009. 33648890 PMC8261838

[90] Ponnudurai T, Meuwissen JH, Leeuwenberg AD, Verhave JP, Lensen AH. 1982. The production of mature gametocytes of *Plasmodium falciparum* in continuous cultures of different isolates infective to mosquitoes. Trans R Soc Trop Med Hyg 76:242–250. 10.1016/0035-9203(82)90289-9. 7048650

[91] Mok S, Stokes BH, Gnadig NF, Ross LS, Yeo T, Amaratunga C, Allman E, Solyakov L, Bottrill AR, Tripathi J, Fairhurst RM, Llinas M, Bozdech Z, Tobin AB, Fidock DA. 2021. Artemisinin-resistant K13 mutations rewire *Plasmodium falciparum*'s intra-erythrocytic metabolic program to enhance survival. Nat Commun 12:530. 10.1038/s41467-020-20805-w. 33483501 PMC7822823

[92] de Hoon MJ, Imoto S, Nolan J, Miyano S. 2004. Open source clustering software. Bioinformatics 20:1453–1454. 10.1093/bioinformatics/bth078. 14871861

[93] Saldanha AJ. 2004. Java Treeview–extensible visualization of microarray data. Bioinformatics 20:3246–3248. 10.1093/bioinformatics/bth349. 15180930

[94] Li H, Handsaker B, Wysoker A, Fennell T, Ruan J, Homer N, Marth G, Abecasis G, Durbin R, 1000 Genome Project Data Processing Subgroup. 2009. The sequence alignment/map format and SAMtools. Bioinformatics 25:2078–2079. 10.1093/bioinformatics/btp352. 19505943 PMC2723002

