# Supplementary material for: Comparative Analysis of Plasmodium falciparum Genotyping via SNP Detection, Microsatellite Profiling, and Whole-Genome Sequencing
Source: Antimicrob Agents Chemother. 2022 Jan 18;66(1):e01163-21. doi: 10.1128/AAC.01163-21 (PMC8765236; doi:10.1128/AAC.01163-21)
Supplement: Supplemental file 1 — Supplemental material. Download AAC.01163-21-s0001.pdf, PDF file, 2.0 MB [file aac.01163-21-s0001.pdf]

SUPPLEMENTAL MATERIAL

Comparative Analysis of *Plasmodium falciparum* Genotyping via SNP Detection, Microsatellite Profiling, and Whole-Genome Sequencing

Mariko Kanai, Tomas Yeo, Victor Asua, Philip J. Rosenthal, David A. Fidock, and Sachel Mok

Table of Contents

FIG S1. Sensitivity test of parasite culture PCR genotyping, in five drug resistance-conferring genes tested in nine mixtures containing different ratios of NF54 and Cam3.II blood stage cultures.

FIG S2. Validation of fragment analysis (FA)- and whole-genome sequencing (WGS)-based microsatellite (MS) genotyping for 12 strains.

FIG S3. Sensitivity test of gel electrophoresis- versus fragment analysis (FA)-based microsatellite (MS) genotyping for four markers using 11 mixtures with different ratios of HB3:Dd2 genomic DNA.

FIG S4. Whole-genome sequencing (WGS)-based microsatellite (MS) genotyping of 15 laboratory strains and sequencing metrics.

TABLE S1. PCR primers and reaction conditions for SNP genotyping by parasite culture PCR and Sanger sequencing.

TABLE S2. PCR primers and reaction conditions for gel electrophoresis and fragment analysis-based microsatellite (MS) genotyping.

TABLE S3. Sanger sequencing results of PCR using simulated fresh whole blood, frozen whole blood, and dried blood spot (DBS) gDNA.

TABLE S4. Single-nucleotide polymorphisms and microsatellite sizes of dried blood spots of Ugandan parasite samples and controls.

TABLE S5. KEL1/PLA1/PfPailin (KelPP) co-lineage status assayed in ten *P. falciparum* strains.

SUPPLEMENTAL METHODS

Parasite culture PCR protocol for SNP genotyping.

Gel electrophoresis- and fragment analysis-based protocol for microsatellite genotyping.

Plasmepsin 2 (pm2) copy number quantification by multiplexed TaqMan qPCR protocol.

SUPPLEMENTAL REFERENCES

Figure S1

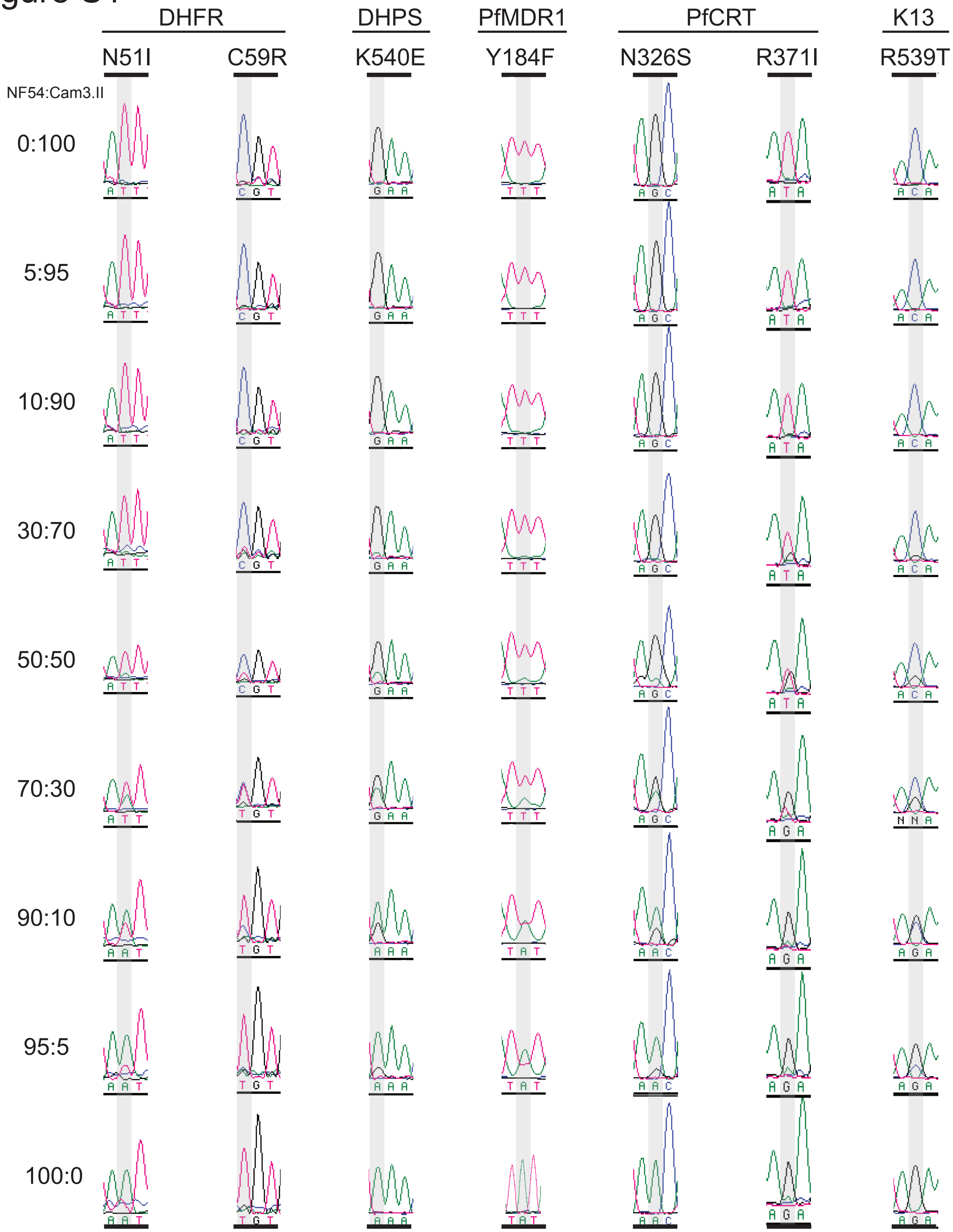

FIG S1 Sensitivity test of parasite RBC culture PCR genotyping, in five drug resistance-conferring genes tested in nine mixtures containing different ratios of NF54 and Cam3.II blood stage cultures. Electropherograms showing the genotypes assessed for these mixtures in three independent occasions.

Figure S2

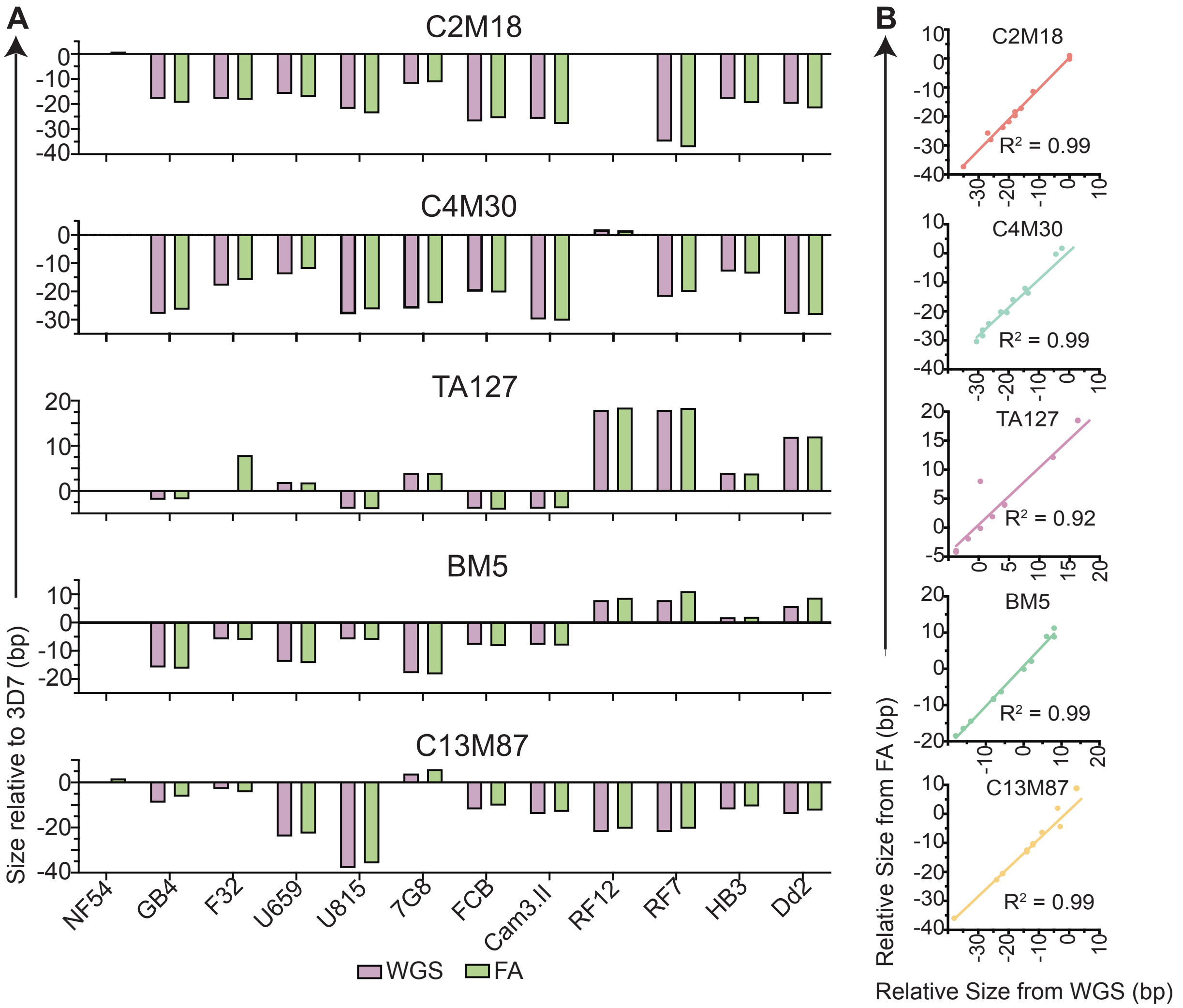

FIG S2 Validation of fragment analysis (FA)- and whole-genome sequencing (WGS)-based microsatellite (MS) genotyping for 12 strains. (A) FA- versus WGS-derived MS size for each strain at the five selected markers relative to 3D7 (see Table 2). (B) Scatterplots showing high correlation (R^2 values) between FA- and WGS-derived MS sizes.

Figure S3

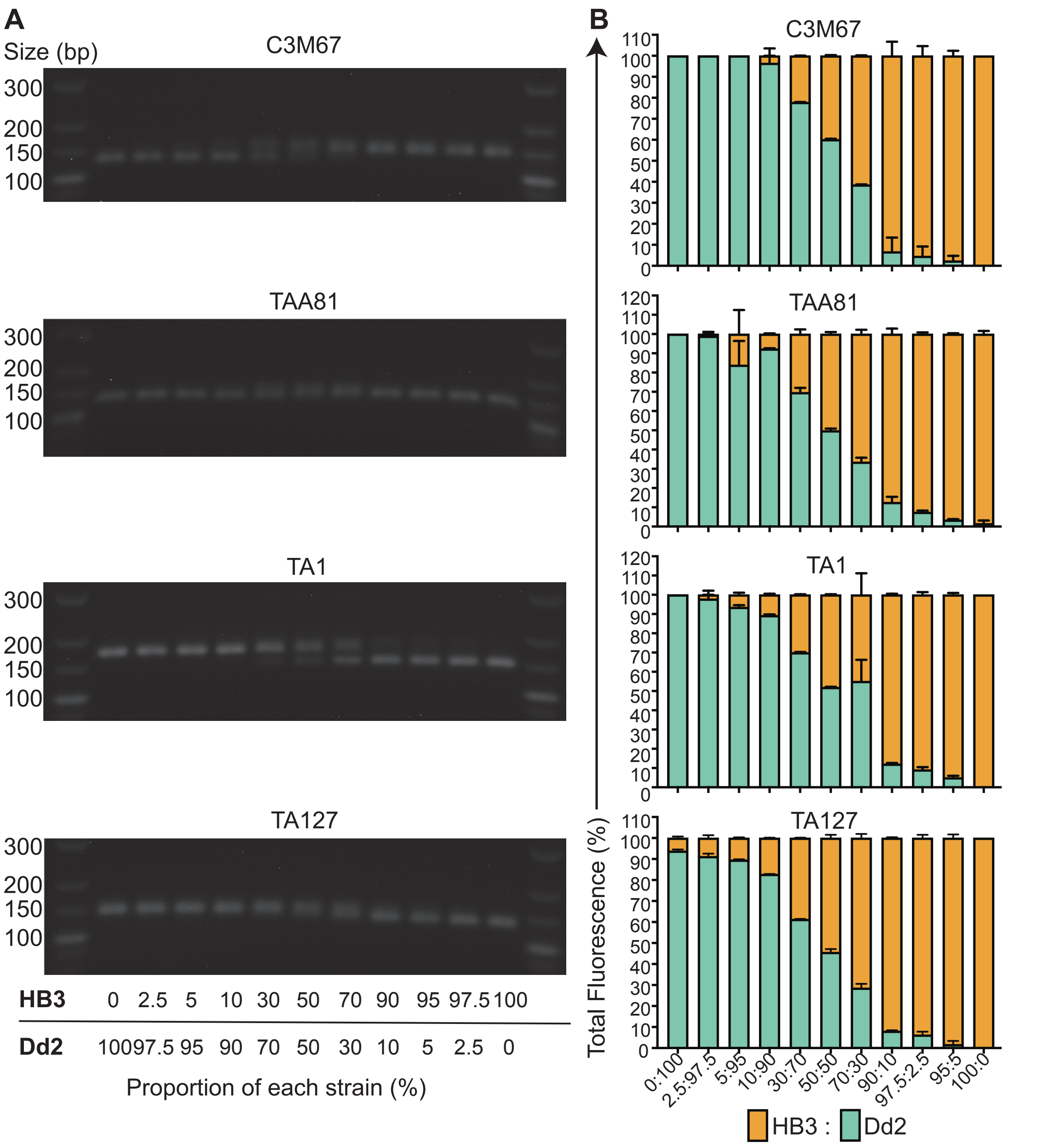

FIG S3 Sensitivity test of gel electrophoresis- versus fragment analysis (FA)-based microsatellite (MS) genotyping for these four markers using 11 mixtures with different ratios of HB3:Dd2 genomic DNA. (A) Gel electrophoresis images of PCR products corresponding to HB3 or Dd2 MS sizes in each mixture. (B) Bar charts represent the average proportion of HB3 or Dd2 fragment sizes in each mixture determined from FA in two independent experiments. Error bars represent SEM values.

Figure S4

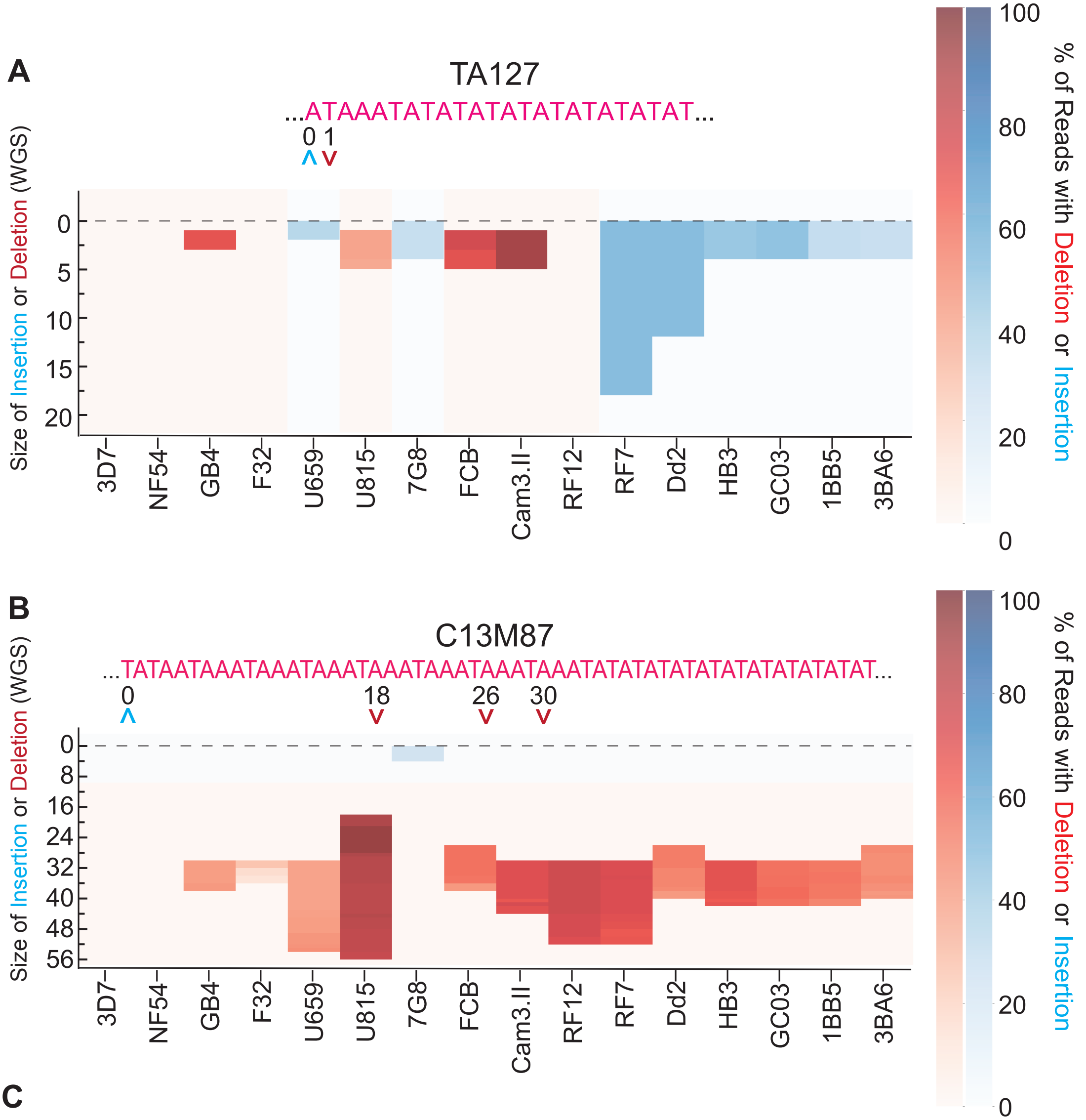

C

Whole-genome sequencing metrics for the 15 *P. falciparum* strains analyzed herein

Dataset		3D7	NF54	GB4	F32	U659	U815	7G8	FCB	Cam3.II	RF12	RF7	Dd2	HB3	GC03	1BB5	3BA6
Sequencer		MiSeq			NextSeq			MiSeq									
Read length (bp)		2 × 300			2 × 150			2 × 300									
Depth of coverage	Mean	47.2	50.6	54.6	44.4	47.4	37.9	51.9	36.8	81.3	77.8	75.8	43.8	79.9	76.9	88.8	72.9
	5×	96.7%	99.9%	96.1%	93.3%	95.2%	94.9%	97.3%	94.6%	92.7%	95.0%	92.6%	90.6%	94.1%	94.2%	94.3%	94.0%
	10×	95.8%	99.8%	95.1%	91.4%	93.5%	92.6%	96.3%	92.4%	59.4%	71.2%	56.6%	48.8%	86.5%	85.0%	88.6%	83.6%
	30×	79.9%	92.5%	85.3%	78.6%	82.4%	72.2%	89.3%	65.6%	55.2%	54.3%	55.3%	56.5%	56.2%	56.3%	56.3%	56.3%

FIG S4 Whole-genome sequencing (WGS)-based microsatellite (MS) genotyping of 15 laboratory strains and sequencing metrics. Heatmaps are shown for two MS markers, (A) TA127 and (B) C13M87, indicating the % of reads harboring an insertion (blue) or deletion (red) sequence for each strain relative to 3D7. The MS sequence for 3D7 (pink) and the position at which the insertion or deletion occurred within the MS fragment window are shown. (C) WGS metrics for the *P. falciparum* strains analyzed in this study.

TABLE S1 PCR primers and reaction conditions for SNP genotyping by parasite culture PCR and Sanger sequencing

Drug Resistance-Associated Gene[a]	Exon	Drug Resistance-Associated Change		Sanger Sequencing Primer		Forward Primer		Reverse Primer		PCR Product Size (kb)	35 cycles[b]					
											Denaturation		Annealing		Extension	
		Amino Acid	Nucleotide	Sequence (5'→3')	Lab Primer ID	Sequence (5'→3')	Lab Primer ID	Sequence (5'→3')	Lab Primer ID		Temp (ºC)	Time (min)	Temp (ºC)	Time (min)	Temp (ºC)	Time (min)
dihydrofolate reductase (*dhfr*)	1	N51I<br>C59R<br>S108N/T<br>I164L	aAt/aTt<br>Tgt/Cgt<br>aGc/aAc/aCc<br>Ata/Tta	ACAAGTCTGCGACGTTTTCGATATTTATG	p7818	ACAAGTCTGCGACGTTTTCGATATTTATG	p7818	AGTATATACATCGCTAACAGA	p7819	0.64	95	0:30	52	1:00	62	1:00
dihydropteroate synthase (*dhps*)	2	S436A/F/C<br>A437G	TCt/Gct/tTt/tGt<br>gCt/gGt	ATGCTTAAATGATATGATACCCG	p7924	ATGCTTAAATGATATGATACCCG	p7924	CATGTAATTCATCTGAAACATCC	p7872	0.97	95	0:30	52	1:00	62	1:00
		K540E/N<br>A581G<br>A613S/T	AaA/Gaa/aaT<br>gCg/gGg<br>Gcc/Tcc/Acc	CATGTAATTCATCTGAAACATCC	p7872											
multidrug resistance protein 1 (*pfmdr1*)	1	N86Y<br>Y184F	Aat/Tat<br>tAt/tTt	TTATTTGATTTTGTGTTGAAAGATGG	p792	TTATTTGATTTTGTGTTGAAAGATGG	p792	TTTGGTAATGTTTCTCCATCATCA	p286	1.14	95	0:30	54	0:30	62	2:30
		G293D	gGt/gAt	CATAAACATACTAATAAGTACACC	p7920											
		N86Y<br>Y184F	Aat/Tat<br>tAt/tTt	TGTATGTGCTGTATTATCAGGA	p7820[c]	TGTATGTGCTGTATTATCAGGA	p7820	CTCTTCTATAATGGACATGGTA	p7821	0.60	95	0:30	52	0:40	62	1:00
		S1034C<br>N1042D	Agt/Tgt<br>Aat/Gat	TCATTTCATAATGCTCTTTCTGG	p7923	TCATTTCATAATGCTCTTTCTGG	p7923	GCAGCAAACTTACTAACACG	p7823	1.13	95	0:30	52	1:00	62	1:00
		D1246Y	Gat/Tat	GCAGCAAACTTACTAACACG	p7823											
chloroquine resistance transporter (*pfcrt*)	2	C72S<br>M74I<br>N75E<br>K76T<br>T93S<br>H97L/Y	Tgt/Agt<br>atG/atT<br>AaT/GaA<br>aAa/aCa<br>Aca/Tca<br>CAc/cTc/Tac	CCCTTGTCGACCTTAACAGATGGCTC	p3519	ATGAAATTCGCAAGTAAAAAAAAATAATCAAAAAAAATTCAAGC	p7011	CCAATCATTTGTTCTTCAATTAAGTATTCCTATTAATATGT	p8402	0.86	95	0:30	52	0:30	62	1:00
	3	F145I	Ttc/Atc	GAACGACACCGAAGCTTTA	p7947											
	4	I218F<br>A220S	Att/Ttt<br>Gcc/Tcc	ATTTACTCCTTTTTAGATATCACTTA	p7868	ATTTACTCCTTTTTAGATATCACTTA	p7868	TTATATTTTTTAAAAACTATTTCCCTTG	p7869	0.35	95	0:30	48	0:30	62	0:30
	6	Q271E	Caa/Gaa	ATTTCTTATAGGCTATGGTATCC	p3805	ATTTCTTATAGGCTATGGTATCC	p3805	CCAAGAATAAACATGCGAAACC	p3806	0.25	95	0:30	50	0:30	62	0:45
	9	N326S/D	AAc/aGc/Gac	CCCATATTTATTTCCTCTTGTATG	p7719	TATGTGATGATTGTGACGGAGC	p7950	TTATAGAACCAAATAGGTAGCC	p7949	0.87	95	0:30	55	0:30	62	0:45
	10	M343L	Atg/Ttg	ATTCTTAGGCTAAGAATTTAAAG	p7989											
		C350R<br>G353V<br>I356T/L	Tgt/Cgt<br>gGt/gTt<br>ATa/aCa/Tta	ATGTAACCATATAATTTTTC	p7956											
	11	R371I	aGa/aTa	AAATGTTTATGATGGTACAACG	p7979											
kelch-13 (*k13*)	1	R539T<br>C580Y	aGa/aCa<br>tGt/tAt	CGGAGTGACCAAATCTGGGA	p6175	GGGAATCTGGTGGTAACAGC	p6176	CGGAGTGACCAAATCTGGGA	p6175	2.10	95	0:30	58	0:30	62	1:45

[a]Gene IDs are *dhfr* (PF3D7_0417200), *dhps* (PF3D7_0810800), *pfmdr1* (PF3D7_0523000), *pfcrt* (PF3D7_0709000), and *k13* (PF3D7_1343700).

[b]For all PCR reactions, the initial denaturation step is 95ºC for 3 min, and final extension is at 62ºC for 3 min for all conditions.

[c]p7820 and p7821 work better than p792 and p286 for sequencing *pfmdr1* in clinical isolates and whole blood.

TABLE S2 PCR primers and reaction conditions for gel electrophoresis and fragment analysis-based microsatellite (MS) genotyping

MS Marker	Chr.	Location (Kb)	Distinct from 3D7	Multiplex Group[b]	Forward Primer			Reverse Primer		30 cycles[c]						MS Repeat Sequence (5'→3'; 3D7; Variable Region Bolded) including Fwd/Rev Primers[d]	Description of MS Marker Location
					Unlabeled Primer		5' Modification	Unlabeled Primer		Denaturatio		Annealing		Extension			
					Sequence (5'→3')	Lab Primer	Lab Primer	Sequence (5'→3')	Lab Primer	Temp (ºC)	Time (min)	Temp (ºC)	Time (min)	Temp (ºC)	Time (min)		
C2M18[a]	2	206	GB4, F32, U659, U815, 7G8, FCB, Cam3.II, RF7, HB3, Dd2	A	CAAGTCGGTTGTATGATAT	p7198	p7734 (6-FAM)	TGTCGATTGGATAATGTTTG	p7199	98	0:20	54	0:15	65	0:15	CAAGTCGGTTGTATGATAT**(AT)$_{21}$AAAAT(ATA)$_{2}$CAAGTC(AT)$_{3}$A(AT)$_{4}$A(AT)$_{4}$(TATA)$_{2}$TTATACAT(TA)$_{5}$T(TA)$_{3}$TTTATTT**CAAACATTATCCAATCGACA	3'UTR of PF3D7_0204700 (hexose transporter)
C3M67	3	804	GB4, U815, 7G8, FCB, Cam3.II, RF12, RF7, HB3, Dd2		GTGTACCTCAAATTATTACC	p7200	p7800 (6-FAM)	TTTGAAATGTTTATGGGAAC	p7201	98	0:20	54	0:15	65	0:15	GTGTACCTCAAATTATTACC**AATAC(TA)$_{3}$TTACTATG(TA)$_{20}$TGTAATATAG(TT)$_{19}$TAAA**GTTCCCATAAACATTTCAAA	3' UTR of PF3D7_0319200 (*ccr4-4*)
C4M30[a]	4	1082	GB4, F32, U659, U815, 7G8, FCB, Cam3.II, RF7, HB3, Dd2	B	ATTGATGCTTTGTCTAATTAG	p7202	p7735 (6-FAM)	ATGACAAAACATGGTATGTA	p7203	98	0:20	50	0:15	65	0:15	ATTGATGCTTTGTCTAATTAG**AGTATATG(AT)$_{8}$(AAA)$_{4}$AT(AA)$_{3}$TA(AAAT)$_{2}$(AA)$_{3}$T(AA)$_{4}$T(AA)$_{3}$T(AA)$_{3}$TAAA(AT)$_{23}$T(TTT)$_{2}$(TA)$_{3}$TTCATA**TACATACCATGTTTTGTCAT	3' UTR of PF3D7_0424000 (*Plasmodium* exported protein, unknown function) and 3'UTR of PF3D7_0424100 (reticulocyte binding homologue 5)
TAA81 / TA81	5	1214	GB4, F32, U659, U815, 7G8, FCB, Cam3.II, RF12, RF7, HB3, Dd2		TTTCACACAACACAGGATT	p7208	p7801 (6-FAM)	TGGACAAATGGGAAAGGATA	p7209	98	0:20	57	0:15	65	0:15	TTTCACACAACACAGGATT**ATTACCATCACCATTAATGTTATCATTCCTTG(TAT)$_{30}$GTTTGATTTGGTT(ATC)$_{2}$TT**TATCCTTTCCCATTTGTCCA	Exon 1 of PF3D7_0529800 (conserved *Plasmodium* protein, unknown function)
TA1	6	900	GB4, F32, U659, U815, 7G8, FCB, Cam3.II, RF12, RF7, HB3, Dd2		TTTTATCTTCATCCCCAC	p7210	p7802 (6-FAM)	CCGTCATAAGTGCAGAGC	p7211	98	0:20	54	0:15	65	0:15	TTTTATCTTCATCCCCAC**A(AAT)$_{2}$(ATTATT)$_{7}$ATT(ATC)$_{4}$(ATTATT)$_{3}$A(TCA)$_{4}$TTA(TT)$_{4}$(ATT)$_{2}$A(TCA)$_{2}$TTA(TT)$_{4}$(ATT)$_{3}$TTCGTTTATAT**GCTCTGCACTTATGACGG	Exon of PF3D7_0622100 (conserved *Plasmodium* protein, unknown function)
TA127[a]	8	1160	RF12, RF7, Dd2	B	GCTTTATAAAAATAACACACC	p7222	p7739 (6-FAM); p7792 (VIC)	TAAAAAACACTCAGTTTGAG	p7223	98	0:20	50	0:15	65	0:15	GCTTTATAAAAATAACACACC**(TA)$_{4}$A(AT)$_{14}$TATATACATTTCTATCTTTA(TTCT)$_{2}$TTTTA(TT)$_{6}$AT**CTCAAACTGAGTGTTTTTTA	3'UTR of PF3D7_0826700 (receptor for activated C kinase)
BM5[a]	8	1224	GB4, F32, U659, U815, 7G8, FCB, Cam3.II, RF12, RF7, Dd2	A	GAAAGTAGATTGTAGTATTTA	p7220	p7738 (6-FAM); p7791 (NED)	TACACATGAATGATTTAATCA	p7221	98	0:20	50	0:15	65	0:15	GAAAGTAGATTGTAGTATTTA**A(TT)$_{3}$TATTA(AT)$_{3}$GGATC(TAT)$_{2}$TTTATTCAC(AT)$_{17}$GTG(TA)$_{4}$TTTTA(TT)$_{3}$CCTTT**TGATTAAATCATTCATGTGTA	Exon-3'UTR of PF3D7_0828400 (mitochondrial inner membrane protein)
C13M87[a]	13	1912	GB4, U659, U815, FCB, Cam3.II, RF12, RF7, HB3, Dd2	B	GATGAGAGAAGGTTAAAATA	p7236	p7740 (6-FAM); p7793 (NED)	CTTCAACACATCTATGGATA	p7237	98	0:20	50	0:15	65	0:15	GATGAGAGAAGGTTAAAAT**(ATA)$_{2}$(AATA)$_{7}$(TATATA)$_{8}$T(TATG)$_{2}$TGTGC(AT)$_{3}$T**TATCCATAGATGTGTTGAAG	Exon5-Intron5-Exon6 of PF3D7_1347800 (leucine-rich repeat protein)

[a]Indicate the 5 MS markers selected for genotyping laboratory strains.

[b]For the multiplex groups, VIC or NED primers were used for TA127, BM5, and C13M87.

[c]For all PCR reactions, the initial denaturation step is 95ºC for 3 min, followed by the 30 cycles as indicated. These cycles are followed by a 65ºC final extension step for 3 min. FAM, fluorescein amidite.

[d]MS repeat motifs were identified using the BioPHP Microsatellite Repeats Finder (1) with the following settings: length of repeated sequence = 2-6bp, min. # of repeats = 2, min. length of tandem repeat = 5, allowed % of mismatches = 0.

TABLE S3 Sanger sequencing results of PCR using simulated fresh whole blood, frozen whole blood, and dried blood spot (DBS) gDNA

Simulated fresh blood		*dhfr*[a,b]				*dhps*				*mdr1*		*pfcrt*					*k13*	
	Parasitemia	N51I	C59R	S108N	I164L	S436A	A437G	K540E	A581G	N86Y	Y184F	C72S	M74I	N75E	K76T	H97Y	R539T	C580Y
NF54	2.00%	✓	✓	✓	✓	✓	✓	✓	✓	✓	✓	✓	✓	✓	✓	✓	✓	✓
	0.40%	✓	✓	✓	✓	✓	✓	✓	✓	✓	✓	nd	nd	nd	nd	nd	✓	✓
	0.08%	nd	nd	nd	nd	✓	✓	✓	✓	nd	✓	nd	nd	nd	nd	nd	✓	✓
	0.02%	nd	nd	nd	nd	nd	nd	✓	nd	nd	nd	nd	nd	nd	nd	nd	nd	nd
Cam3.II	2.00%	✓	✓	✓	✓	✓	✓	✓	✓	✓	✓	✓	✓	✓	✓	✓	✓	✓
	0.40%	nd	nd	nd	nd	✓	✓	✓	✓	✓	✓	✓	✓	✓	✓	✓	✓	✓
	0.08%	nd	nd	nd	nd	✓	✓	✓	✓	✓	✓	nd	nd	nd	nd	nd	✓	✓
	0.02%	nd	nd	nd	nd	✓	✓	✓	✓	✓	✓	nd	nd	nd	nd	nd	nd	nd

Simulated frozen blood		*dhfr*				*dhps*				*mdr1*		*pfcrt*					*k13*	
	Parasitemia	N51I	C59R	S108N	I164L	S436A	A437G	K540E	A581G	N86Y	Y184F	C72S	M74I	N75E	K76T	H97Y	R539T	C580Y
NF54	2.00%	✓	✓	✓	✓	✓	✓	✓	✓	✓	✓	✓	✓	✓	✓	✓	✓	✓
	0.40%	✓	✓	✓	✓	✓	✓	✓	✓	✓	✓	nd	nd	nd	nd	nd	✓	✓
	0.08%	nd	nd	nd	nd	nd	nd	✓	✓	✓	✓	nd	nd	nd	nd	nd	nd	nd
	0.02%	nd	nd	nd	nd	nd	nd	nd	nd	✓	✓	nd	nd	nd	nd	nd	nd	nd
Cam3.II	2.00%	✓	✓	✓	✓	✓	✓	✓	✓	✓	✓	✓	✓	✓	✓	✓	✓	✓
	0.40%	✓	✓	✓	✓	✓	✓	✓	✓	✓	✓	nd	nd	nd	nd	nd	✓	✓
	0.08%	nd	nd	nd	nd	nd	nd	✓	✓	✓	✓	nd	nd	nd	nd	nd	nd	nd
	0.02%	nd	nd	nd	nd	nd	nd	nd	nd	✓	✓	nd	nd	nd	nd	nd	✓	✓

Simulated DBS gDNA		*dhfr*				*dhps*				*mdr1*		*pfcrt*					*k13*	
	Parasitemia	N51I	C59R	S108N	I164L	S436A	A437G	K540E	A581G	N86Y	Y184F	C72S	M74I	N75E	K76T	H97Y	R539T	C580Y
NF54	2.00%	✓	✓	✓	✓	✓	✓	✓	✓	✓	✓	✓	✓	✓	✓	✓	nd	nd
	0.40%	✓	✓	✓	✓	✓	✓	✓	✓	✓	✓	✓	✓	✓	✓	✓	✓	✓
	0.08%	✓	✓	✓	✓	✓	✓	✓	✓	✓	✓	✓	✓	✓	✓	✓	✓	✓
	0.02%	nd	nd	nd	nd	nd	nd	nd	nd	nd	nd	✓	nd	nd	nd	✓	✓	✓
Cam3.II	2.00%	✓	✓	✓	✓	✓	✓	✓	✓	✓	✓	✓	✓	✓	✓	✓	nd	nd
	0.40%	✓	✓	✓	✓	✓	✓	✓	✓	✓	✓	✓	✓	✓	✓	✓	nd	✓
	0.08%	✓	✓	✓	✓	✓	✓	✓	✓	✓	✓	✓	✓	✓	✓	✓	nd	✓
	0.02%	✓	✓	✓	✓	nd	nd	nd	nd	✓	✓	✓	✓	✓	✓	✓	nd	✓

[a]√ = expected allele detected; nd = allele not detected.

[b]Gene IDs are *dhfr* (PF3D7_0417200), *dhps* (PF3D7_0810800), *pfmdr1* (PF3D7_0523000), *pfcrt* (PF3D7_0709000), and *k13* (PF3D7_1343700).

TABLE S4 Single-nucleotide polymorphisms and microsatellite sizes of dried blood spots of Ugandan parasite samples and controls

	Drug Resistance-Associated Gene and Change in Amino Acid[a,b]																	
	dhfr				*dhps*					*mdr1*[c]		*pfcrt*					*k13*	
**Samples**[d]	N51I	C59R	S108N	I164L	I431V	S436A	A437G	K540E	A581G	N86Y	Y184F	C72S	M74I	N75E	K76T	H97Y	R539T	C580Y
**NF54**	WT	WT	WT	WT	WT	WT	WT	WT	WT	WT	WT	WT	WT	WT	WT	WT	WT	WT
**TO-04-01**	MUT	MUT	MUT	WT	nd	nd	nd	MUT	WT	WT	WT	WT	WT	WT	WT	WT	WT	WT
**TO-04-03**	MUT	MUT	MUT	WT	WT	WT	WT	MUT	WT	WT	WT	WT	WT	WT	WT	WT	WT	WT
**TO-04-04**	MUT	MUT	MUT	WT	WT	WT	WT	MUT	WT	WT	MUT	WT	WT	WT	WT	WT	WT	WT
**TO-04-05**	MUT	MUT	MUT	WT	WT	WT	WT	MUT	WT	WT	MIX	WT	WT	WT	WT	WT	WT	WT
**TO-04-06**	MUT	MUT	MUT	MIX	WT	WT	WT	MUT	nd	nd	nd	WT	WT	WT	WT	WT	WT	WT
**TO-04-07**	MUT	MUT	MUT	WT	WT	WT	WT	MUT	WT	WT	MUT	WT	WT	WT	WT	WT	WT	WT
**TO-04-08**	MUT	MIX	MUT	WT	WT	WT	WT	MUT	WT	WT	WT	WT	WT	WT	WT	WT	WT	WT
**TO-04-09**	MUT	MUT	MUT	WT	WT	WT	WT	MUT	nd	nd	WT	WT	WT	WT	WT	WT	WT	WT
**TO-04-11**	MUT	MIX	MUT	WT	WT	WT	WT	MUT	nd	nd	nd	WT	WT	WT	WT	WT	WT	WT
**TO-04-17**	MUT	MUT	MUT	WT	WT	WT	WT	MUT	WT	WT	MIX	WT	WT	WT	WT	WT	WT	WT
**TO-04-19**	MUT	MUT	MUT	WT	WT	WT	WT	MUT	WT	WT	WT	WT	WT	WT	WT	WT	WT	WT
**TO-04-20**	MUT	MIX	MUT	WT	WT	WT	WT	MUT	WT	WT	MUT	WT	WT	WT	WT	WT	WT	WT
**TO-04-21**	MUT	WT	MUT	WT	WT	WT	WT	MUT	WT	WT	MUT	WT	WT	WT	WT	WT	WT	WT
**TO-04-22**	MUT	MUT	MUT	WT	WT	WT	WT	MUT	WT	WT	MUT	WT	MUT	MUT	MUT	WT	WT	WT
**TO-04-23**	MUT	MUT	MUT	WT	WT	WT	WT	MUT	WT	WT	MUT	WT	WT	WT	WT	WT	WT	WT
**TO-04-24**	MUT	MUT	MUT	WT	WT	WT	WT	MUT	WT	WT	WT	WT	WT	WT	WT	WT	WT	WT
**U659**	MUT	MUT	MUT	WT	WT	WT	WT	MUT	WT	WT	MUT	WT	MUT	MUT	MUT	WT	WT	WT

	Microsatellite Sizes Relative to NF54 (bp)														
	**BM5**			**C13M87**			**C2M18**			**C4M30**			**TA127**		
**Samples**[d]	Major Peak	% of Major	Minor Peaks	Major Peak	% of Major	Minor Peaks	Major Peak	% of Major	Minor Peaks	Major Peak	% of Major	Minor Peaks	Major Peak	% of Major	Minor Peaks
**Absolute Size of NF54**	143			141			159			187			125		
**TO-04-01**	+27	62%	+3, -14	-28	100%	--	-17	100%	--	-24	100%	--	+4	100%	--
**TO-04-03**	-6	100%	--	-24	100%	--	-24	100%	--	-28	100%	--	nd	--	--
**TO-04-04**	+7	71%	-18	nd	--	--	-22	100%	--	-26	100%	--	+4	100%	--
**TO-04-05**	+13	72%	-12	nd	--	--	-13	100%	--	-28	100%	--	-6	100%	--
**TO-04-06**	nd	--	--	-22	100%	--	nd	100%	--	-28	100%	--	nd	--	--
**TO-04-07**	-4	100%	--	-24	100%	--	nd	100%	--	-28	100%	--	nd	--	--
**TO-04-08**	+5	100%	--	-5	100%	--	-19	100%	--	-28	100%	--	0	100%	--
**TO-04-09**	-4	100%	--	nd	--	--	nd	100%	--	-28	100%	--	nd	--	--
**TO-04-11**	nd	--	--	nd	--	--	-20	100%	--	-28	100%	--	nd	--	--
**TO-04-17**	+17	67%	-8	-22	100%	--	-22	100%	--	+12	71%	-28	0	100%	--
**TO-04-19**	-2	57%	-8	-26	100%	--	-24	100%	--	-28	100%	--	0	100%	--
**TO-04-20**	+15	74%	-10	-15	72%	-2	-28	100%	--	-28	100%	--	0	100%	--
**TO-04-21**	+15	77%	-10	-15	100%	--	-24	100%	--	-26	100%	--	0	100%	--
**TO-04-22**	-16	100%	--	-17	100%	--	-22	100%	--	-17	100%	--	-4	100%	--
**TO-04-23**	+9	58%	-16	-10	100%	--	-10	86%	-1	-28	100%	--	nd	--	--
**TO-04-24**	+19	62%	-4	-26	100%	--	-24	100%	--	-11	100%	--	+4	100%	--
**U659**	-14	100%	--	-22	100%	--	-17	100%	--	-14	100%	--	+2	100%	--

[a]Gene IDs are *dhfr* (PF3D7_0417200), *dhps* (PF3D7_0810800), *pfmdr1* (PF3D7_0523000), *pfcrt* (PF3D7_0709000), and *k13* (PF3D7_1343700).
[b]WT = wild-type; MUT = mutant; MIX = both wild-type and mutant peaks detected; ND = not detected.
[c]PCR primers p7820 and p7821, and sequencing primer p7820 worked better for detecting *pfmdr1* alleles than p792 and p286 in DBS samples.
[d]NF54 and U659 samples used gDNA purified from cultures. All other samples were gDNA extracted from dried blood spots collected from 16 Ugandan patients harboring symptomatic *P. falciparum* infections.

TABLE S5 KEL1/PLA1/PfPailin (KelPP) co-lineage status assayed in ten *P. falciparum* strains

Parasite Name	Provider	Origin	Year	K13[a]	PfCRT[a,b]	*k13*-flanking Microsatellite Size Relative to 3D7						Plasmepsin 2[a] Copy No.
						-50kb	-31.9kb	-6.36kb	-0.15kb	+8.6kb	+31.5kb	
NF54/3D7	--	Africa	1979	WT	WT	0	0	0	0	0	0	1
U659	P. Rosenthal	Uganda	2007	WT	GB4	-9	-4	-6	-18	14	3	1
S170 Clone 1H1	L. Musset	French Guiana	2018	WT	7G8+C350R	-4	-2	-3	-30	-14	-3	≥2 (PLA1)
Dd2	T. Wellems	Cambodia	1980	WT	Dd2	-6	-6	0	-30	4	-3	1
Cam2 (IPC 3445)	D. Ménard	Pailin, W. Cambodia	2010	C580Y (KEL1)	Dd2	-6	-6	0	-30	12	-3	1
PH0482-C Clone E11	R. Fairhurst	Pursat, W. Cambodia	2010	C580Y (KEL1)	Dd2	-6	-6	0	-30	16	-3	1
PH0212-C Clone C11	R. Fairhurst	Pursat, W. Cambodia	2010	C580Y (KEL1)	Dd2	-6	-6	0	-30	16	-3	1
KH001-061	S. Volkman	Pursat, W. Cambodia	2011	C580Y (KEL1)	Dd2	-6	-6	0	-30	17	-3	1
RF7 (PH1008-C)[c]	R. Fairhurst	Pursat, W. Cambodia	2012	C580Y (KEL1)	Dd2+M343L	-6	-6	0	-30	16	-3	≥2 (PLA1)
Cam3.II (RF967 / PH0306-C)	R. Fairhurst	Pursat, W. Cambodia	2010	R539T (KEL3)	Dd2	-6	-6	0	-30	-10	6	1

[a]Gene IDs are *k13* (PF3D7_1343700), *pfcrt* (PF3D7_0709000), and *plasmepsin 2* (PF3D7_1408000).

[b]The *pfcrt* haplotype for Dd2: M74I N75E K76T A220S Q271E N326S I356T R371I; GB4: M74I N75E K76T A220S Q271E R371I; 7G8: C72S K76T A220S N326D I356L.

[c]RF7 is the only KEL1/PLA1/PfPailin haplotype identified herein.

SUPPLEMENTAL METHODS

Parasite culture PCR protocol for SNP genotyping

Required reagents:

- ddH_2O
- Bioline 2× MyTaq Blood-PCR Mix (Cat. No. BIO-25054)
- Forward, Reverse, and Sequencing Primers (diluted to 5 μM)
- 3 μL of ≥ 1% parasitemia *P. falciparum*-infected packed RBCs (pRBCs)
- PCR tubes
- PCR thermal cycler
- Centrifuge
- PCR purification kit
- Sanger sequencer and sequence analyzer software

Method:

1. Prepare the PCR reaction master mix as follows, referencing the primer combination listed in **Table S1**:

Reagent	Per Reaction (μL)
ddH_2O	15
2× MyTaq Blood-PCR Mix	20
Fwd Primer (5 μM each)	1
Rev Primer (5 μM each)	1

   a. Add 37 μL of the reaction master mix to a reaction tube.
   b. Add 3 μL of *P. falciparum*-infected pRBCs and mix with a pipette.
   c. Briefly centrifuge reaction mixture for 2-3 seconds.
2. Run the PCR reaction according to the conditions outlined in **Table S1**.
3. Spin the PCR reaction for 1 min at 8000g.
4. Making sure to avoid the blood pellet, transfer 30 μL of the supernatant to a new PCR tube.
   (*Can pause here and leave samples in a 4ºC fridge overnight*).
5. Run 7 μL of the supernatant on a 0.8% gel for 40 min at 120V.
6. PCR-purify 20 μL of the supernatant and send the PCR product for Sanger sequencing with the appropriate sequencing primer.
7. Identify the amino acid codon at the desired position using a sequence analyzer software.

Gel electrophoresis- and fragment analysis-based protocol for microsatellite genotyping

Required reagents:

- Roche KAPA HiFi HotStart Ready Mix (2×) (Cat. No. KK2601)
- ddH_2O
- Forward, Reverse, and Sequencing Primers (diluted to 10 µM)
  - *For PCR reactions for fragment analysis, the Fwd or Rev primer must be fluorescently labeled.
- PCR tubes or 96-well plate
- PCR thermal cycler
- Centrifuge
- Gel electrophoresis:
  - Agarose
  - TBE Buffer
  - Invitrogen™ Ultra Low Range Ladder (Cat. No. 10597012) (includes 6× TrackIt Cyan/Yellow Loading Buffer)
  - Electrophoresis tank and power supply
  - Gel imager
- Fragment analysis:
  - Applied Biosystems™ ExoSAP-IT™ *Express* PCR Product Cleanup Reagent (Cat. No. 75001.1.ML)
  - DNA sizing software (e.g. Thermo Fisher Scientific Peak Scanner)
- Reference parasite strains (e.g. 3D7, genetic cross parents) with known marker sizes should be included as internal controls on the gel and in each fragment analysis run, respectively.

Method:

1. Prepare the PCR reaction master mix as follows, referencing the primer combination listed in **Table S2**:

Reagent	Per Reaction (µL)
ddH_2O	3.6
KAPA HiFi HotStart Ready Mix (2×)	7.5
Fwd Primer (10µM)	0.45
Rev Primer (10µM)	0.45

   a. Add 12 µL of the master mix to a PCR tube or well.
   b. Add 3 µL of gDNA template (4 ng/µL).
   c. Seal the tube or plate, and mix by vortexing for 2-3 seconds.
   d. Briefly spin reaction mixture by centrifugation for 2-3 sec (tube) or 1000rpm x 1 min (plate).

2. Run PCR according to the conditions outlined in **Table S2**.
   (*Can pause after PCR and store samples at -20ºC*).

Gel Electrophoresis:

3. Run 5 µL of PCR product on a 2% agarose gel at 100V for 1.5-2 h, ensuring that all strains for each marker fit into a single comb within one gel.
4. Run 6 µL of the 1× Ultra Low Range Ladder on both ends of the gel for band size identification.
5. Visualize the PCR products with a gel imager.
6. Identify the microsatellite size for each strain using the reference strain as a comparator.
    a. Note: if more than one band is observed for the sample, this may indicate a polyclonal sample.

Fragment Analysis (FA):

1. If multiplexing, mix PCR products according to the following ratios:

Marker	Group for Multiplexing	Label	Mixing Ratio Post-PCR
C2M18	A	6-FAM (Blue)	2
BM5		NED/ATTO550 (Yellow)	1
C4M30	B	6-FAM (Blue)	1
TA127		VIC/ATTO532 (Green)	1
C13M87		NED/ATTO550 (Yellow)	1

2. Clean up PCR product by adding 2 µL of ExoSAP-IT™ *Express* PCR Product Cleanup Reagent to 5 µL of PCR product in a new tube or well.
3. Vortex for 2-3 seconds to ensure mixing.
4. Spin down for 2-3 sec (tube) or 1000rpm × 1 min (plate).
5. Incubate at 37ºC for 4 min and 80ºC for 1 min to inactivate the enzyme in a thermocycler.
6. Dilute sample 10× by adding 2 µL of cleaned-up product with 18 µL of ddH_20.
7. Send 20 µL of reaction for FA using the Liz500 ladder.
8. Analyze microsatellite size using a DNA sizing software.
    a. Restrict sizes to 100-300 bp, peak threshold to 500 for desired color (e.g. blue for FAM) and 50 for others not in use (e.g. red).
    b. Export data as an Excel file, and sort by sample name (and color if multiplexing), and then by peak area.
    c. The size of the peak with the largest area indicates the dominant MS size.
    d. Obtain absolute and relative MS sizes (compared to reference strain such as NF54).
    e. Note: If analyzing genetic cross progeny, use the MS sizes in the parental samples as a reference for the progeny. The MS sizes in the progeny should match the MS size in either parent.
    f. If detecting potential contamination by another strain
        i. If (minor peak area / (major + minor peak area) *100% > 10%) at the same marker for the same sample, flag as a mixture. However, if <10% mix seen in multiple markers for a sample, repeat PCR+FA to verify clonal purity.

Plasmepsin 2 (pm2) copy number quantification by multiplexed TaqMan qPCR protocol

Required reagents:

- NanoDrop or Qubit
- Ice
- ddH_2O
- Bio-Rad SsoAdvanced Universal Probes Supermix (2×) (Cat. No. 1725282)
- Dual-Labeled TaqMan Probes (DLPs; diluted to 10 μM)
    - *pm2*: 5'-6-FAM-CAACATTTGATGGTATCCTTGGTTTAGGATGGA-MGB-Eclipse-3'
    - *β-tubulin*: 5'-HEX-TAGCACATGCCGTTAAATATCTTCCATGTCT-BHQ1-3'
- Primers (diluted to 10 μM)
    - *pm2* Fwd: GGATTCGAACCAACTTATACTGC
    - *pm2* Rev: AATTGGATCTACTGAACCTATTGATAA
    - *β-tubulin* Fwd: TGATGTGCGCAAGTGATCC
    - *β-tubulin* Rev: TCCTTTGTGGACATTCTTCCTC
- Gene fragment standards of *pm2*: *β-tubulin* mixed at 1:1, 2:1, 3:1, 4:1, and 5:1 molar ratios as described in (2).
- Applied Biosystems MicroAmp EnduraPlate Optical 96-well Fast Clear Reaction Plate with Barcode (Cat. No. 4483485)
- Applied Biosystems Optical Adhesive Covers (Cat. No. 4360954)
- Centrifuge
- Real-Time PCR System

Method:

1. Use a NanoDrop or Qubit to quantify template DNA on the same day as the qPCR run, and adjust to 4 ng/μL if necessary.
2. Thaw Sso 2× mix, probes, primers, and standards on ice and protected from light for 20 minutes.
3. Prepare the PCR reaction master mix as follows:

Reagent	Volume (μL) per reaction	Final Concentration
ddH_2O	5.02	
Sso mix (2×)	10	1×
pm2 Fwd+Rev Primer Mix (10μM each)	0.66	0.33 μM
pm2 TaqMan probe	0.33	0.16 μM
β-tubulin Fwd+Rev Primer Mix (10μM each)	0.66	0.33 μM
β-tubulin TaqMan probe	0.33	0.16 μM

   a. Get a clear qPCR plate and label sections according to each sample.
   b. Add 17 μL of the reaction master mix to each tube or well.
   c. Add 3 μL of template DNA (4 ng/μL) or ddH_2O (no template control, NTC) in triplicate for each sample and mix well by pipetting.
   d. Include as a *pm2* copy 1 control: 3D7 or NF54
   e. Include as *pm2* copy 1-5 controls: gene fragment standards of *pm2*: *β-tubulin* mixed at 1:1, 2:1, 3:1, 4:1, and 5:1 molar ratios.

4. Seal the plate using a plate seal applicator.
5. Spin down for 1000rpm × 1 min.
6. Run the qPCR program in a Real-Time PCR system as follows:

Cycles	Step	Temperature	Duration
1	Denaturation	95°C	3 min
40	Denaturation	95°C	20 s
	Annealing, Extension, Detection	58°C	40 s

7. Quantify copy number by using the standard curve method for relative quantification:

$$ratio = \frac{(1.8293)^{\Delta Ct_{(pfpm2)}}}{(1.8531)^{\Delta Ct_{(pf\beta-tubulin)}}}$$

$$\Delta Ct(pm2) = avg.Ct(pm2\ in\ 3D7\ reference) - avg.Ct(pm2\ in\ sample)$$

$$\Delta Ct(\beta - tubulin) = avg.Ct\ (\beta - tubulin\ in\ 3D7\ reference) - avg.Ct\ (\beta - tubulin\ in\ sample)$$

a. 1.8293 and 1.8531 are the primer efficiencies for *pm2* and *β-tubulin*, respectively. Primer efficiencies were determined using qPCR analysis of serially-diluted gDNA as detailed in (3).

SUPPLEMENTAL REFERENCES

1. Bikandi J. 2006. BioPHP - Microsatellite Repeats Finder (version 2). http://insilico.ehu.es/mini_tools/microsatellites/. Retrieved 12 April 2021. *{Code and/or software.}*
2. Witkowski B, Duru V, Khim N, Ross LS, Saintpierre B, Beghain J, Chy S, Kim S, Ke S, Kloeung N, Eam R, Khean C, Ken M, Loch K, Bouillon A, Domergue A, Ma L, Bouchier C, Leang R, Huy R, Nuel G, Barale JC, Legrand E, Ringwald P, Fidock DA, Mercereau-Puijalon O, Ariey F, Menard D. 2017. A surrogate marker of piperaquine-resistant *Plasmodium falciparum* malaria: a phenotype-genotype association study. Lancet Infect Dis 17:174-183.
3. Ross LS, Dhingra SK, Mok S, Yeo T, Wicht KJ, Kumpornsin K, Takala-Harrison S, Witkowski B, Fairhurst RM, Ariey F, Menard D, Fidock DA. 2018. Emerging Southeast Asian PfCRT mutations confer *Plasmodium falciparum* resistance to the first-line antimalarial piperaquine. Nat Commun 9:3314.
